# Xerophytic Lichens from Gypsiferous Outcrops of Arid Areas of Andalusia as a Source of Anti-Phytopathogenic Depsides

**DOI:** 10.3390/jof9090887

**Published:** 2023-08-30

**Authors:** Ignacio Fernández-Pastor, Victor González-Menéndez, Kevin Martínez Andrade, Rachel Serrano, Thomas A. Mackenzie, Guillermo Benítez, Manuel Casares-Porcel, Olga Genilloud, Fernando Reyes

**Affiliations:** 1 Fundación MEDINA, Avda. Del Conocimiento 34, Health Sciences Technology Park, 18016 Granada, Spain; ignacio.fernandez@medinaandalucia.es (I.F.-P.); kevin.martinez@medinaandalucia.es (K.M.A.); rachel.serrano@medinaandalucia.es (R.S.); thomas.mackenzie@medinaandalucia.es (T.A.M.); olga.genilloud@medinaandalucia.es (O.G.); 2 Department of Botany, Faculty of Pharmacy, Cartuja Campus, University of Granada, 18071 Granada, Spain; gbcruz@ugr.es (G.B.); mcasares@ugr.es (M.C.-P.)

**Keywords:** xerophytic lichens, antifungal activity, biopesticides, fungal phytopathogens, dereplication, MS/MS molecular networking, depsides

## Abstract

In a survey to evaluate the potential of lichens associated with gypsum areas as sources of new antifungal metabolites, six species of lichens were collected in the gypsum outcrops of the Sorbas Desert (*Diploschistes ocellatus* and *Seirophora lacunosa*) and the Tabernas Desert (*Cladonia foliacea*, *Acarospora placodiformis*, *Squamarina lentigera* and *Xanthoparmelia pokornyi*) in southern Spain. Raw lichen acetone extracts were tested against a panel of seven phytopathogenic fungi, including *Botrytis cinerea*, *Colletotrichum acutatum*, *Fusarium oxysporum* f.sp *cubense* TR4, *Fusarium ploriferaum*, *Magnaporthe grisea*, *Verticillium dahliae* and *Zymoseptoria tritici*. Active extracts of *Cladonia foliacea*, *Xanthoparmelia pokornyi* and *Squamarina lentigera* were analyzed by HPLC-MS/MS and Molecular Networking to identify possible metabolites responsible for the antifungal activity. A total of ten depside-like metabolites were identified by MS/MS dereplication and NMR experiments, of which one was a new derivative of fumaroprotocetraric acid. The compounds without previously described biological activity were purified and tested against the panel of fungal phytopathogens. Herein, the antifungal activity against fungal phytopathogens of 4′-O-methylpaludosic acid, divaricatic acid and stenosporic acid is reported for the first time. Stenosporic and divaricatic acids displayed a broad antifungal spectrum against seven relevant fungal phytopathogens in a micromolar range, including the extremely resistant fungus *F. oxysporum* f. sp. *cubense* Tropical Race 4 (TR4). 4′-O-methylpaludosic acid exhibited specific antifungal activity against the wheat pathogen *Z. tritici*, with an IC50 of 38.87 µg/mL (87.1 µM) in the absorbance-based assay and 24.88 µg/mL (55.52 µM) in the fluorescence-based assay.

## 1. Introduction

Within all plant pathogens, the fungi kingdom is a highly diverse group that represents a devastating threat to plant health in agriculture, resulting in a negative economic impact worldwide with every single outbreak [1]. Genetic plasticity allows fungi to quickly invade new hosts, as well as to develop resistance to traditional fungicidal substances used against these phytopathogens [2]. Even with modern agriculture, fungal epidemics still occur in plants and generate substantial losses in terms of food production yields. For instance, back in 2016, the first known outbreak caused by the wheat blast fungus *Magnaporthe oryzae* in Asia devastated more than 15,000 hectares of wheat crops in Bangladesh. The available treatments could not contain the infection of the fungus, which strikes wheat heads, making it difficult for fungicides to reach the pathogen [3]. Another example is *Fusarium oxysporum* f. sp. *cubense* Tropical Race 4 (TR4), an extremely resistant fungus that can persist in soil or on decaying host plant debris for up to 30 years in the form of chlamydospores and cause an outbreak in a Cavendish banana plantation in North Queensland, Australia, in March 2015 [4,5]. In Europe, a new variety of stem-rust fungus attacked Sicilian crops back in 2016, destroying ten thousand hectares of crops and alarming researchers since this new strain displayed unusually high infection capabilities against plants resistant to disease [6].

Addressing this issue, the European Parliament developed Directive 2009/128/EC, related to the sustainable use of pesticides, and Regulation (EC) No. 1107/2009 on the marketing of phytosanitary products, both promoting the replacement of traditional chemical agents for phytosanitary treatments and other alternatives when possible. This last regulation was recently modified in Regulation (EU) 2017/1432, which established the use of novel low-risk natural substances as starting products for the development of new treatments, even when their half-life in the soil exceeds 60 days. These recent regulations open a new niche in the biodiscovery of substances with high-added value in the plant health market. This work is focused on the use of endemic gypsiferous lichens from southern Spain as a possible source for these substances, based on preliminary bioactivity results.

Lichens are symbiotic organisms born from the association of fungi and microalgae, which has allowed them to develop a complex metabolism with exceptional adaptation capabilities, allowing them to survive in hostile and desolated environments from the poles to the deserts [7]. Among the mechanisms deployed for the adaptation of these organisms to harsh conditions, their response to oxidative stress [8], desiccation tolerance [9] and potential to produce secondary metabolites with antimicrobial properties can be highlighted [10,11,12]. Lichens inhabiting gypsiferous fields are particularly rare species due to their chemical nature [13] and extremely hostile conditions [14] in such environments, and they have mainly been studied from a botanical point of view during the last forty years [15,16,17,18,19]. For this reason, gypsiferous lichens are a poorly exploited source for the potential biodiscovery of new secondary metabolites with several applications.

Among the metabolites widely known to be produced by lichens, depsides are a class of polyphenols structurally featuring two or more monocyclic aromatic moieties linked by an ester bond. This class of compounds has previously shown antioxidant activity [20,21], antiproliferative activity on cancer cells [22,23], antibiofilm activity [24], antimicrobial activities on both bacteria and fungi [25,26], and some other relevant bioactivities [27]. Compounds belonging to this structural class were already detected in the preliminary chemical analysis of the gypsiferous lichen extracts. 

The objective of this research was the screening of xerophytic lichen samples from the Andalusian gypsiferous outcrops (Tabernas and Sorbas, Almeria province), followed by the isolation and identification of the bioactive compounds responsible for the anti-phytopathogenic activity. 

## 2. Materials and Methods

### 2.1. General

Extracts were analyzed by HPLC-UV-ESI-TOF on an Agilent 1200 RR, coupled to a Bruker maXis time-of-flight spectrometer with electrospray ionization, as reported by Martin et al. [28]. Mass spectra were collected as full scans from 50 *m/z* to 2000 *m/z*. Data were analyzed using the platform available at Fundación MEDINA [29] and compared with the data available in Medina’s In-House internal database and the Dictionary of Natural Products database. LC-UV-LRMS analyses were performed on an Agilent 1260 Infinity II (Agilent Technologies, Santa Clara, CA, USA) equipped with a single quadrupole LC-MS system. Mono- and bidimensional Nuclear Magnetic Resonance (NMR) spectra were recorded at 297K on a Bruker Avance III spectrometer (500 and 125 MHz for ^1^H and ^13^C, respectively) equipped with a 1.7 mm TCI MicroCryoProbeTM (Bruker Biospin, Fällanden, Switzerland). Chemical shifts from ^1^H and ^13^C were reported in parts per million using the signals of the residual solvents as internal reference (δ_H_ 2.50 and δ_C_ 39.52 ppm for DMSO-*d*_6_).

### 2.2. Lichen Material

**Figure 1:**
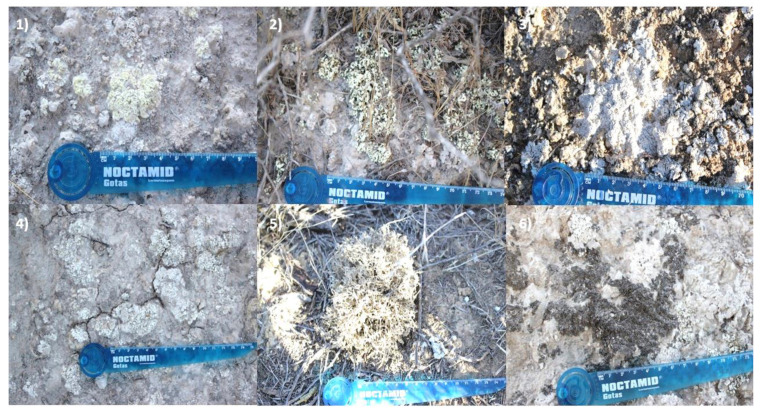
Xerophytic lichens from gypsiferous outcrops: (**1**) *Acarospora placodiiformis*; (**2**) *Cladonia foliacea* f. *convoluta*; (**3**) *Diploschistes ocellatus* var. *almeriensis*; (**4**) *Squamarina lentigera*; (**5**) *Seirophora lacunosa* and (**6**) *Xanthoparmelia pokornyi*.

Samples of *Diploschistes ocellatus* var *almeriensis* and *Seirophora lacunosa* were collected from gypsiferous outcrops in Sorbas, Almeria, Spain, in October of 2020. Samples of *Acarospora placodiiformis*, *Cladonia foliacea*, *Squamarina lentigera* and *Xanthoparmelia pokornyi* were collected in Venta de los yesos, Tabernas Desert, Almeria, Spain, in October of 2020 (Table 1, Figure 1). 

The lichens were taxonomically identified by one of us (M.C.-P.), and reference samples were preserved in the University of Granada herbarium (GDA), with voucher codes provided in Table 1.

### 2.3. Extraction 

The lichen samples were washed with distilled water and dried before their extraction. For the extraction, two grams of each sample were weighed and divided into two equal portions. One of the portions was extracted with 10 mL of acetone, and the other with 10 mL of a mixture of dichloromethane and methanol (1:1). All samples were extracted overnight by maceration. The resulting crude extracts were filtered, and the organic phase was evaporated under a nitrogen stream. Then, 5 mg of each sample was resuspended in 50 µL of DMSO to prepare the stock solution for the antifungal assays (100 mg/mL). Inactive extracts were not studied/analyzed further. 

### 2.4. LC-MS/MS and Feature-Based Molecular Networking Analysis

LC-MS/MS acquisition was performed as previously described in the general section. Ionization of the eluting solvent was obtained using the standard maXis ESI source, adjusted to a drying gas flow of 11 L/min at 200 °C and a nebulizer pressure of 40 psig. The capillary voltage was set to 4000 V. Mass spectra were collected from 150 *m/z* to 2000 *m/z* in positive mode. The mass spectrometer was operated in data-dependent positive ion mode, automatically switching between full scan MS and MS/MS acquisitions. Full scan MS spectra (*m/z* 50–2000) were acquired in the TOF, and the top six most intense ions in a particular scan were fragmented using collision-induced dissociation (CID).

MS/MS spectra were converted to the mzXML file format by using Bruker Data Analysis software. The mzXML file was further processed with MZmine 2.539 following the described processing steps: mass detection, chromatogram builder, chromatogram deconvolution, isotopic peaks grouper, feature alignment, gap filling, filtering and direct submit/export for GNPS [30]. The final processed file was then used for the online analysis on the GNPS platform (https://gnps.ucsd.edu/ accessed on 25 July 2023), and Cytoscape 3.6.1 was used to visualize the generated molecular networks. FBMN data are available from the following link: https://gnps.ucsd.edu/ProteoSAFe/status.jsp?task=6cafd8eec333427a81ccb5497bee4b7a accessed on 25 July 2023.

### 2.5. Crude Extract Fractionation

A total of 80 mg of each lichen extract was fractionated through a Gilson semi-automated HPLC-DAD system by creating a gradient on a column Zorbax Preparative SB-C8 (PrepHT Zorbax SB-C8 21.2 × 250 mm, 7 μm). Solvents selected were H_2_O (Solvent A) and CH_3_CN (Solvent B), both acidified with 0.1% trifluoroacetic acid (TFA) to increase the resolution of the peaks. The flow rate was set at 20 mL/min, and the wavelengths selected for DAD detection were 210 nm and 256 nm. The gradient of the method proceeded as follows: 1 min at 5%B (t = 1 min), 35 min from 5%B to 100%B (t = 36 min), 7 min at 100%B (t = 43 min), 2 min to initial conditions (t = 45 min). The main peaks observed on the generated chromatogram were collected and analyzed through both HPLC-UV-ESI-TOF and HPLC-UV-LRMS to dereplicate and verify purity. 

### 2.6. Purification of Compounds

Fractions containing either more than one compound or a compound with impurities were submitted to semipreparative HPLC using the same conditions as reported for the fractionation of the crude extracts (Section 2.5). The differences rely on the flow rate (3.6 mL/min for semipreparative purification) and the gradient, which was different for each specific case. Gradients were built as follows: 1 min at chosen initial conditions, 35 min from initial %B to final %B, 7 min washing at 100%B and 2 min to restore initial conditions. A gradient annotated as 5–100 B refers to 5 as initial %B and 100 as final %B. 8′-Hydroxyfumarprotocetraric acid was purified by 20–40 B gradient, divaricatic acid purification was performed using a 45–75 B gradient, stenosporic acid was purified by 50–100 B gradient; 4′-O-Methylpaludosic acid and sekikaic were obtained purely from the crude extract fractionation. 

The compounds purified from the raw lichen extracts were analyzed by HPLC-UV-LRMS, HPLC-UV-ESI-TOF and 1D/2D-NMR to confirm their identity. A compound was considered pure when both the area of the peak on the UV trace at 210 nm represented 90% of all the observed peaks, and no relevant impurities were observable in its ^1^H NMR spectrum.

### 2.7. Anti-Phytopathogen Bioassays

The anti-fungal activity of xerophytic lichens was evaluated against seven relevant phyto-pathogenic strains: *Botrytis cinerea* B05.10, *Colletotrichum acutatum* CF-137177, *Fusarium oxysporum* f.sp. *cubense* CBS 102025, *Fusarium proliferatum* CBS 115.97, *Magnaporthe grisea* CF-105765, *Verticillium dahliae* CBS 717.96 and *Zymoseptoria tritici* CBS 115943.

Liquid-based assay was performed by incubating samples (lichen extracts or isolated compounds) with conidia solutions (1 × 10^6^ conidia/mL) of target microorganisms during a specific incubation time for each fungal strain (24–120 h) at 25 °C, using RPMI modified medium. The antifungal activities were scored using absorbance differences at 600 nm, between the final and the initial incubation times, as indicator of the mycelia development and resazurin as an oxidation-reduction indicator of cell viability [31].

Lichen extracts were tested by dissolving them in 100% DMSO at 2 mg/mL. Pure compounds were tested in two-fold dilutions (10 points), starting at 160 µg/mL dissolved in 100% DMSO, and 2 µL of each point was dispensed in each assay plate per triplicate. IC_50_ values were calculated based on the dose–response curves obtained from both readouts, corresponding to the dose/concentration that induced half-maximum responses, as indicative of potency measure. The results obtained were analyzed using Genedata Screener software (Genedata Inc., Basel, Switzerland) [31]. 

### 2.8. Antitumoral Activity Assays

Pure compounds were also tested in MTT assays against human cancer cell lines to see if they had cytotoxic properties and/or affected the liver. Samples dissolved in 100% DMSO were assayed in two-fold dilution 10-point curves starting at 40 µg/mL (1:200 assay dilution reaching a final DMSO percentage of 0.5%), in triplicate for 72 h. For the cytotoxicity against cancer cell lines study we used A549 (ATCC^®^ CCL-185™, lung carcinoma), A2058 (ATCC^®^ CRL-11147^TM^, melanoma), MCF7 (ATCC^®^ HTB-22^TM^, breast adenocarcinoma) and MIA PaCa-2 (ATCC^®^ CRL-1420^TM^, pancreas carcinoma), whereas for the liver cytotoxicity study we worked with HepG2 cells (ATCC^®^ HB-8065^TM^, hepatocellular carcinoma). The resulting data was analyzed using Genedata Screener software (Genedata Inc., Basel, Switzerland). 

## 3. Results

### 3.1. Antifungal Activity of Lichen Extracts

Characterization of the antifungal properties of the six lichen extracts (*A. placodiformis*, *C. foliacea*, *D. ocellatus*, *S. lacunose*, *S. lentigera* and *X. pokornyi*) was carried out, testing them for fungal growth inhibition (absorbance) and cell viability (fluorescence), against a panel of seven fungal phytopathogens. After this evaluation, extracts from the lichens *C. foliacea*, *S. lentigera* and *X. pokornyi* showed total inhibition of *Z. tritici* based on absorbance and fluorescence readouts. 

To identify the active metabolites responsible for *Z. tritici* inhibition, semipreparative HPLC fractions of extracts from the three lichens were conducted. Several active fractions against *Z. tritici* were selected for HPLC-MS analysis and further purification.

### 3.2. Identification of Main Active Peaks from the Raw Extracts

Antifungal active fractions were analyzed by HPLC-MS for the dereplication of known lichen metabolites. The dereplication of these molecules was then achieved by HPLC-UV-ESI-TOF analysis and comparison with the data available in the In-House database and commercial Chapman and Hall Dictionary of Natural Products, as reported in methods. Dereplication was based on retention time, biological source and the molecular formula (calculated from the *m/z* peaks). Confirmation of the identity of the non-dereplicated compounds was performed after HPLC purification by ESI-TOF and NMR Analysis (Table 2). Those compounds without reported biological activity were repurified and tested against the fungal phytopathogens panel described in the methods section. 

### 3.3. Feature-Based Molecular Networking of Lichen Crude Extracts

**Figure 2:**
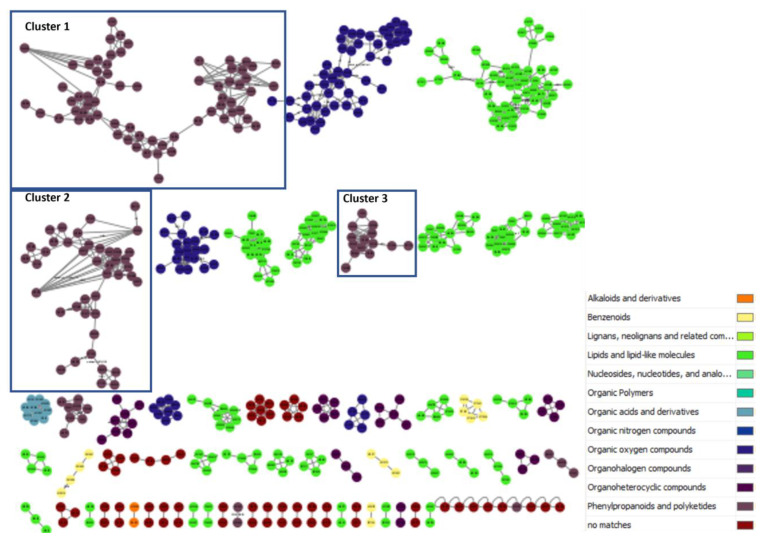
Main clusters from the molecular network of lichen crude extracts.

**Figure 3:**
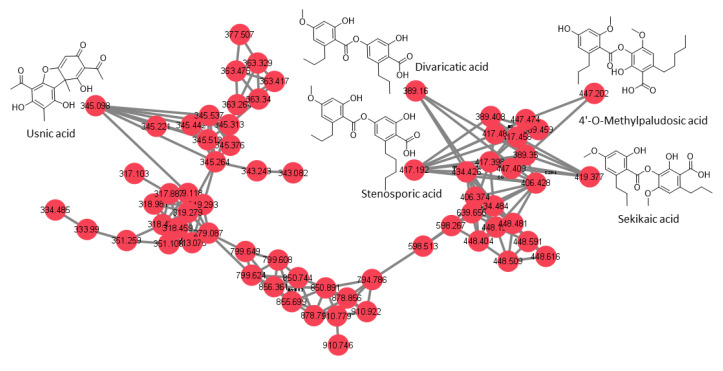
Cluster 1 of the depsides family and usnic acid.

**Figure 4:**
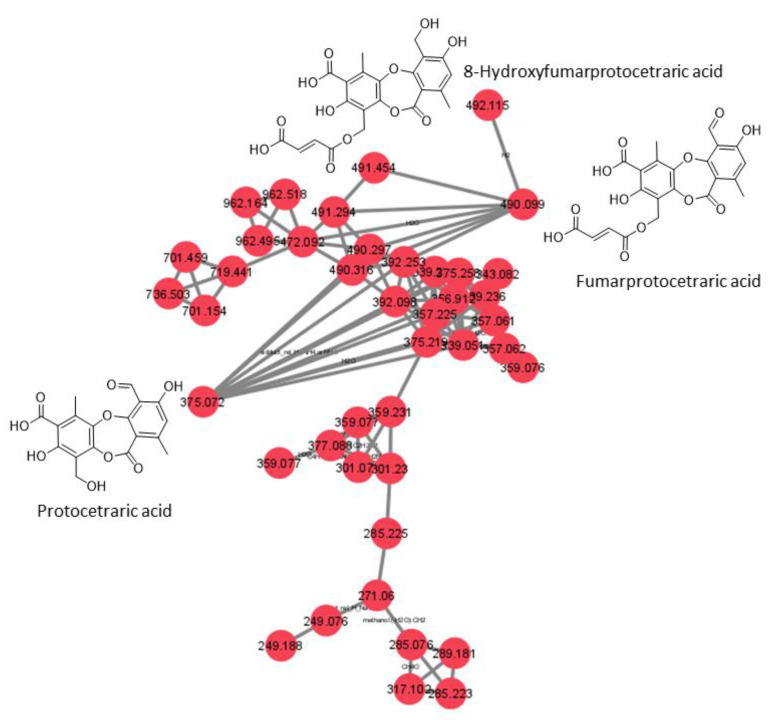
Cluster 2 of the depsidone family.

**Figure 5:**
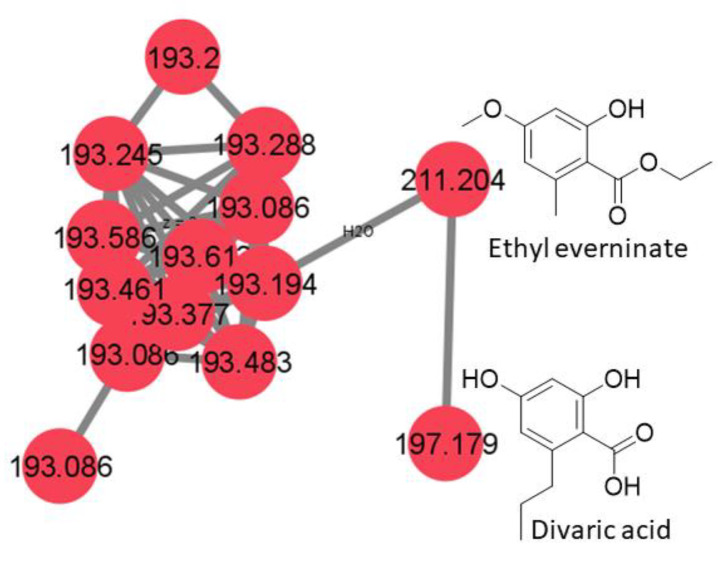
Cluster 3 of the depside monomers family.

An approach combining feature-based molecular networking with bioassay-guided fractionation allowed the detection, isolation and identification of the metabolites responsible for the antifungal activity. The active crude extracts produced by maceration of raw lichenic biomass in acetone were analyzed by HPLC-MS/MS to generate a molecular network to compare samples and to investigate the chemical diversity by dereplication of known compounds. The main clusters of the molecular network obtained for the active lichenic extracts from the Tabernas Desert are shown in Figure 2, in which each node represents a parent ion of an MS/MS spectrum. According to the legend, the color code refers to the different super-classes of metabolites present in the cluster. The occurrence of big clusters associated with phenylpropanoids and polyketides indicates the possible presence of depsides and depsidones, characteristic molecules previously reported from lichens (Figure 3, Figure 4 and Figure 5). 

### 3.4. Structural Elucidation of 8′-Hydroxyfumarprotocetraric Acid

**Figure 6:**
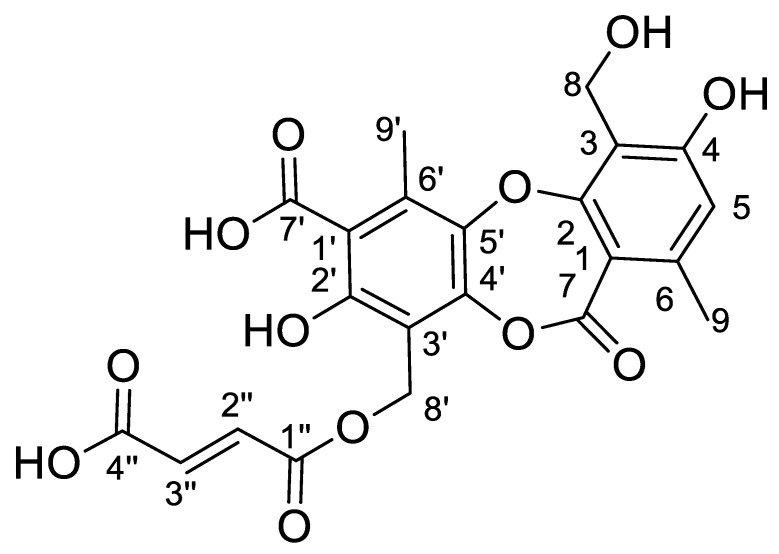
Structure of 8′-Hydroxyfumarprotocetraric acid.

8′-Hydroxyfumarprotocetraric acid (Figure 6) was obtained as a white amorphous solid from the acetone extract of *C. foliacea*. Its molecular formula, C_22_H_18_O_12,_ was deduced from its (+)-ESI-TOF analysis ([M + NH_4_]^+^: 492.1135, Δ −0.2 ppm). The ^13^C NMR spectrum of the compound (Table 3) showed twenty-two carbon signals in total, including four carbonyl carbons at δ_C_ 162.5 (C7), 172.3 (C7′), 164.7 (C1″) and 166.1 (C4″), twelve sp^2^ aromatic carbons from the depsidone core at δ_C_ 111.7, 161.3, 115.6, 161.1, 115.1, 144.6, 110.3, 158.6, 112.2, 147.7, 142.4 and 134.8, two olefinic signals from the fumaric acid moiety at δ_C_ 132.8 and 133.7 and four sp^3^ carbon signals. Its ^1^H spectrum exhibited two characteristic doublet signals from the hydrogens of a fumaric acid moiety δ_H_ 6.73 (H2″) and 6.72 (H3″) ppm, J = 15.5 Hz; one aromatic singlet from the depsidone core δ_H_ 6.64 (H5), two oxygenated methylene singlet signals δ_H_ 5.41 (H8′) and 4.96 ppm (H8) and two methyl singlet signals δ_H_ 2.84 (H9′) and 2.44 (H9) ppm. The fumaric acid moiety was confirmed by the HMBC correlation between H2″ with olefinic C3″ and two carboxylic carbons C1″ and C4″, as well as cross-peaks between H3″ with olefinic C2″, and carboxylic C1″ and C4″. The connection between the dicarboxylic acid moiety with the depsidone core is evidenced by the HMBC signals of hydroxylated *sp*^3^ methylene signal H8′ with carboxylic C1″, H8′ share cross-peaks with C2′, C3′ and C4′ aromatic sp^2^ carbons. Additionally, the methyl singlet H9′ showed correlations with C4′, C5′, C6′ and C7′, leading to the identification of one of the phenolic acid rings. On the other hand, a cross-peak of the singlet aromatic H5 with the sp^2^ aromatic carbons C3, C4 and C6, and with sp^3^ C8 and C9, together with the correlation of methyl singlet H9 with aromatic C1, C5, C6 and carboxylic C7 and methylene singlet H8 cross-peaks with aromatic C3, C4 and C5, explained the configuration of the second phenolic ring. These NMR data share similarities with fumaroprotocetratic spectroscopic data published [32]. 

### 3.5. Anti-Phytopathogenic Activity Characterization of Pure Compounds 

A total of five pure compounds (Figure 7) were obtained from the active HPLC fractions: (1) 8-hydroxyfumarprotocetraric acid from *C. foliacea* (2.3 mg); (2) divaricatic acid (14.7 mg); (3) stenosporic acid from *X. pokornyi* (3 mg); (4) sekikaic acid (1.2 mg) and (5) 4′-O-methylpaludosic acid from *S. lentigera* (10.5 mg). All pure compounds were tested against the panel of fungal phytopathogens based on absorbance and fluorescence in vitro assays, and their corresponding IC_50_ values in μg/mL were calculated (Table 4) from their corresponding dose–response curves (Figures S26–S39). 

The most potent compound was stenosporic acid from *X. pokornyi*, which presented an IC_50_ against *Z. tritici* of 7.53 µg/mL (18.1 µM) in the absorbance-based assay and 6.59 µg/mL (15.84 µM) in the fluorescence-based assay. This compound also displayed significant antifungal activity against *B. cinerea*, *C. acutatum*, *M. grisea* and *V. dahliae* but less potency against *Fusarium* spp. Divaricatic acid also presented similar IC_50_ values for *Z. tritici* (12.4 µg/mL (31.9 µM) in the absorbance-based assay and 10.67 µg/mL (27.49 µM) in the fluorescence-based assay) and a similar antifungal activity pattern for the rest of the six fungal phytopathogens (Table 4). 4′-O-methylpaludosic acid only showed antifungal activity against *Z. tritici*, with an IC_50_ of 38.87 µg/mL (87.1 µM) in the absorbance-based assay and 24.88 µg/mL (55.52 µM) in the fluorescence-based assay. 8-hydroxyfumarprotocetraric acid and sekikaic acid did not display any antifungal activities against the seven fungal pathogens at the highest concentration tested of 160 µg/mL. 

Stenosporic acid was described in 1970 as a new depside from the lichen *Ramalina stenospora* collected in Florida, USA [33]. Later, antimicrobial screening using extracts from *X. pokornyi* reported antimicrobial and antiyeast activity of stenosporic acid, but no antifungal activity has been reported for this compound against filamentous fungi, including *Fusarium* spp. [34]. Results using our in vitro HTS platform have allowed us to describe its antifungal activity, including two *Fusarium* spp.

Divaricatic acid has been reported in extracts obtained from several lichen genera, including *Cladonia*, *Flavocetraria*, *Parmeliopsis*, *Ramalia*, *Roccella Lecanora*, *Ophioparma* and others, to have antibacterial, antifungal and antiparasitic activities [25]. The reported MIC for this compound against *B. cinerea* and *F. oxysporum* was 6.26–12.5 mg/mL [35]. Our screening results confirmed the antifungal properties of divaricatic acid but revealed a higher potency of this compound to inhibit the seven phytopathogens tested, with IC_50_ values in the micromolar range, even against the extremely resistant fungus *Fusarium oxysporum* f. sp. *cubense* Tropical Race 4 (TR4).

The presence of 4′-O-methylpaludosic has been reported in extracts from several *Ramalia* species [36], but no biological activity of this compound has been reported to date. This molecule only showed antifungal activity against the wheat pathogen *Z. tritici* with IC_50_ values in the micromolar range, highlighting its potential use as a specific antifungal compound to control the causal agent of *S. tritici* blotch.

### 3.6. Antitumoral Activity of Pure Compounds 

As mentioned above, the five pure compounds obtained were assayed against five human cancer cell lines in an absorbance-based MTT assay, and ED_50_ values were obtained.

As can be observed in Table 5, none of the compounds had significant activity against the cell lines tested. Divaricatic acid was the compound with more activity against all cell lines, except for MIA PaCa-2, reaching an ED_50_ value of 15.12 µg/mL (38.93 µM) against the MCF7 breast cancer cell line.

## 4. Conclusions

Ten compounds from the bioactive lichens *Cladonia foliacea*, *Squamarina lentigera* and *Xanthoparmelia pokornyi* were identified by MS/MS dereplication, molecular networking and NMR experiments, nine of which were already known molecules and one of which was a new derivative of fumaroprotocetraric acid. 

Stenosporic and divaricatic acids, isolated from the xerophytic lichen *X. pokornyi*, displayed a broad antifungal spectrum against seven relevant fungal phytopathogens, including the extremely resistant fungus *F. oxysporum* f. sp. *cubense* Tropical Race 4 (TR4). Furthermore, divaricatic acid also displayed some activity against a panel of human cancer cell lines, although this was not relevant considering that the activity was only present at high concentrations. Therefore, both compounds could represent promising broad-spectrum disease control agents. Moreover, this is the first report on the biological activity of 4′-O-methylpaludosic acid isolated from *S. lentigera*, which exhibited specific antifungal activity against the wheat *pathogen Z. tritici*, the causal agent of *S. tritici* blotch. These results confirmed our initial hypothesis that Xerophytic lichens from gypsiferous outcrops are an important source of metabolites with anti-phytopathogenic activity. 

## Figures and Tables

**Figure 7:**
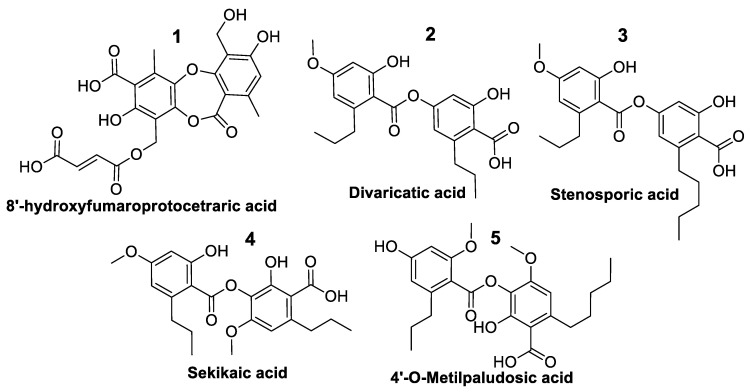
Structure of five pure compounds tested against fungal phytopathogens.

**Table 1:** Xerophytic lichens collected from gypsiferous outcrops.

Lichen	Place	GPS Coordinate	Voucher Code
*Diploschistes ocellatus* (Fr.) Norman var. *almeriensis* Llimona	Sorbas, Almeria	37°5′17″ N/2°5′15″ O	GDA-Lichen 3966
*Seirophora lacunosa* (Rupr.) Fröden	Sorbas, Almeria	37°5′22″ N/2°5′16″ O	GDA-Lichen 3970
*Cladonia foliacea* (Huds.) Willd. f. *convoluta* (Lam.) J. Steiner	Venta de los yesos, Tabernas, Almeria	37°4′60″ N/2°17′37″ O	GDA-Lichen 3965
*Acarospora placodiiformis* (Nyl.) H. Olivier	Venta de los yesos, Tabernas, Almeria	37°4′60″ N/2°17′38″ O	GDA-Lichen 3964
*Xanthoparmelia pokornyi* (Körb.) O. Blanco, A. Crespo, Elix, D. Hawksw. & Lumbsch	Venta de los yesos, Tabernas, Almeria	37°4′60″ N/2°17′37″ O	GDA-Lichen 3969
*Squamarina lentigera* (Weber) Poelt	Venta de los yesos, Tabernas, Almeria	37°4′60″ N/2°17′38″ O	GDA-Lichen 3968

**Table 2:** List of dereplicated lichenic compounds. * HPLC-UV-HRESIMS, NMR or both.

Cluster	t_R_ (min)	Present in Sample	*m/z*	Ion Type	Molecular Formula	Identified Compound *
C1	4.65	*S*. *lentigera*	345.098	[M + H]^+^	C_18_H_16_O_7_	Usnic acid
	5.41	*X*. *pokornyi*	389.160	[M + H]^+^	C_21_H_24_O_7_	Divaricatic acid
	5.53	*S*. *lentigera*	419.377	[M + H]^+^	C_22_H_26_O_8_	Sekikaic acid
	5.96	*X*. *pokornyi*	417.192	[M + H]^+^	C_23_H_28_O_7_	Stenosporic acid
	6.06	*S. lentigera*	447.202	[M + H]^+^	C_24_H_30_O_8_	4′-O-Methylpaludosic acid
C2	3.03	*C. foliacea*	375.072	[M + H]^+^	C_18_H_14_O_9_	Protocetraric acid
	3.13	*C. foliacea*	492.115	[M + NH_4_]^+^	C_22_H_18_O_12_	8′-Hydroxyfumarprotocetraric acid
	4.91	*C. foliacea*	490.099	[M + NH_4_]^+^	C_22_H_16_O_12_	Fumarprotocetraric acid
C3	2.76	*X*. *pokornyi*	197.178	[M + H]^+^	C_10_H_12_O_4_	Divaric acid
	3.90	*X*. *pokornyi*	211.204	[M + H]^+^	C_11_H_14_O_4_	Ethyl everninate

**Table 3:** NMR data of 4-Hydroxyfumarprotocetraric acid in CD_3_OD.

	**CD_3_OD**		**HMBC**
	**^1^H NMR**	**^13^C NMR**	
N°	*d* in ppm, mult, *J* in Hz	*d* in ppm	
1		111.7	
2		161.3	
3		115.6	
4		161.1	
5	6.64, s	115.1	3, 4, 6, 8 and 9
6		144.6	
7		162.5	
8	4.96, s	52.9	3, 4 and 5
9	2.44, s	19.3	1, 5, 6 and 7
1′		110.3	
2′		158.6	
3′		112.2	
4′		147.7	
5′		142.4	
6′		134.8	
7′		172.3	
8′	5.41, s	56.1	2′, 3′, 4′ and 1″
9′	2.84, s	14.1	4′, 5′, 6′ and 7′
1″		164.7	
2″	6.73, d, 15.5	132.8	1″, 3″ and 4″
3″	6.72, d, 15.5	133.7	1″, 2″ and 4″
4″		166.1	

**Table 4:** IC_50_ values in μg/mL for five pure compounds, obtained from xerophytic lichens, against seven important fungal phytopathogens based on absorbance (Abs) and fluorescence (Flu) in vitro assays.

Compound	IC_50_ (µg/mL)													
	*Z. tritici*		*B. cinerea*		*C. acutatum*		*F. oxysporum* TR4		*M. grisea*		*V. dahliae*		*F. proliferatum*	
	Abs	Flu	Abs	Flu	Abs	Flu	Abs	Flu	Abs	Flu	Abs	Flu	Abs	Flu
8′-hydroxy fumarprotocetraric acid	>160	>160	>160	>160	>160	>160	>107	>107	>160	>160	>160	>160	>160	>160
divaricatic acid	**12.4**	**10.67**	**36.87**	**30.29**	**56.84**	**75.75**	**79.00**	**83.28**	**48.85**	**51.85**	**49.38**	**32.11**	**83.13**	**88.07**
stenosporic acid	**7.53**	**6.59**	**38.03**	**38.94**	**69.48**	**98.49**	**77.02**	**93.47**	**36.06**	**41.88**	**51.75**	**27.03**	**94.17**	**103.30**
sekikaic acid	>160	134.6	>160	>160	>160	>160	>107	>107	>160	>160	>160	>160	>160	>160
4′-O-methylpaludosic acid	**38.87**	**24.88**	>160	>160	157.7	106.8	>107	>107	>160	>160	>160	89.78	>160	>160

**Table 5:** ED_50_ values in μg/mL for five pure compounds obtained from xerophytic lichens against five human cancer cell lines.

Compound	ED 50 (µg/mL)				
	A2058	A549	HepG2	MCF7	MIA PaCa-2
8′-hydroxyfumarprotocetraric acid	>40.00	>40.00	>40.00	>40.00	>40.00
divaricatic acid	23.75	26.99	28.41	15.12	>40.00
stenosporic acid	>40.00	>40.00	>40.00	30.13	>40.00
sekikaic acid	>40.00	>40.00	>40.00	>40.00	>40.00
4′-O-methylpaludosic acid	>40.00	>40.00	>40.00	>40.00	>40.00

## Supplementary Materials

The following supporting information can be downloaded at: https://www.mdpi.com/article/10.3390/jof9090887/s1, Figure S1: (a) LC-UV-HRMS chromatogram (UV 210 nm). (b) UV spectrum of 8-Hydroxyfumarprotocetraric acid. (c) HRESIMS-MS(+)-TOF spectra of 8 Hydroxyfumarprotocetraric acid; Figure S2: 1H spectra of 8-Hydroxyfumarprotocetraric acid; Figure S3: HSQC spectra of 8-Hydroxyfumarprotocetraric acid; Figure S4: HMBC spectra of 8-Hydroxyfumarprotocetraric acid; Figure S5: 13C spectra of 8-Hydroxyfumarprotocetraric acid; Figure S6: (a) UV spectrum of fumarprotocetraric acid. (b) HRESIMS-MS(+)-TOF spectra of fumarprotocetraric acid.); Figure S7: 1H spectra of fumaroprotocetraric acid; Figure S8: HSQC spectra of fumaroprotocetraric acid; Figure S9: (a) UV spectrum of divaricatic acid. (b) HRESIMS-MS(+)-TOF spectra of divaricatic acid; Figure S10: 1H spectra of divaricatic acid; Figure S11: HSQC spectra of divaricatic acid; Figure S12: (a) UV spectrum of stenosporic acid. (b) HRESIMS-MS(+)-TOF spectra of stenosporic acid; Figure S13: 1H spectra of stenosporic acid; Figure S14: HSQC spectra of stenosporic acid; Figure S15: (a) UV spectrum of sekikaic acid. (b) HRESIMS-MS(+)-TOF spectra of sekikaic acid; Figure S16: 1H spectra of sekikaic acid; Figure S17: HSQC spectra of sekikaic acid; Figure S18: (a) UV spectrum of 4′-O-Methylpaludosic acid. (b) HRESIMS-MS(+)-TOF spectra of 4′-O-Methylpaludosic acid; Figure S19: 1H spectra of 4′-O-Methylpaludosic acid; Figure S20: HSQC spectra of 4′-O-Methylpaludosic acid; Figure S21: HMBC spectra of 4′-O-Methylpaludosic acid; Figure S22: (a) UV spectrum of ethyl everninate. (b) HRESIMS-MS(+)-TOF spectra of ethyl everninate; Figure S23: (a) UV spectrum of divaric acid. (b) HRESIMS-MS(+)-TOF spectra of divaric acid; Figure S24: (a) UV spectrum of protocetraric acid. (b) HRESIMS-MS(+)-TOF spectra of protocetraric acid; Figure S25: (a) UV spectrum of usnic acid. (b) HRESIMS-MS(+)-TOF spectra of usnic acid; Figure S26: Dose Response Curves, EC50 values and their corresponding 95% confidence limits for compounds 1–5 and Amphotericin B obtained from *Z. tritici* absorbance-based assay; Figure S27: Dose Response Curves, EC50 values and their corresponding 95% confidence limits for compounds 1–5 and Amphotericin B obtained from *Z. tritici* fluorescence-based assay; Figure S28: Dose Response Curves, EC50 values and their corresponding 95% confidence limits for compounds 1–5 and Amphotericin B obtained from B. cinerea absorbance-based assay; Figure S29: Dose Response Curves, EC50 values and their corresponding 95% confidence limits for compounds 1–5 and Amphotericin B obtained from B. cinerea fluorescence-based assay; Figure S30: Dose Response Curves, EC50 values and their corresponding 95% confidence limits for compounds 1–5 and Amphotericin B obtained from C. acutatum absorbance-based assay; Figure S31: Dose Response Curves, EC50 values and their corresponding 95% confidence limits for compounds 1–5 and Amphotericin B obtained from C. acutatum fluorescence-based assay; Figure S32: Dose Response Curves, EC50 values and their corresponding 95% confidence limits for compounds 1–5 and Amphotericin B obtained from *F. oxysporum* TR4 absorbance-based assay; Figure S33: Dose Response Curves, EC50 values and their corresponding 95% confidence limits for compounds 1–5 and Amphotericin B obtained from *F. oxysporum* TR4 fluorescence-based assay; Figure S34: Dose Response Curves, EC50 values and their corresponding 95% confidence limits for compounds 1–5 and Amphotericin B obtained from M. grisea absorbance-based assay; Figure S35: Dose Response Curves, EC50 values and their corresponding 95% confidence limits for compounds 1–5 and Amphotericin B obtained from M. grisea fluorescence-based assay; Figure S36: Dose Response Curves, EC50 values and their corresponding 95% confidence limits for compounds 1–5 and Amphotericin B obtained from V. dahliae absorbance-based assay; Figure S37: Dose Response Curves, EC50 values and their corresponding 95% confidence limits for compounds 1–5 and Amphotericin B obtained from V. dahliae fluorescence-based assay; Figure S38: Dose Response Curves, EC50 values and their corresponding 95% confidence limits for compounds 1–5 and Amphotericin B obtained from F. proliferatum absorbance-based assay; Figure S39: Dose Response Curves, EC50 values and their corresponding 95% confidence limits for compounds 1–5 and Amphotericin B obtained from F. proliferatum fluorescence-based assay.
