# Supplementary material for: Xerophytic Lichens from Gypsiferous Outcrops of Arid Areas of Andalusia as a Source of Anti-Phytopathogenic Depsides
Source: J Fungi (Basel). 2023 Aug 30;9(9):887. doi: 10.3390/jof9090887 (PMC10532656; doi:10.3390/jof9090887)
Supplement: Supplementary file 1 [file jof-09-00887-s001.zip › jof-2551900-supplementary.pdf]

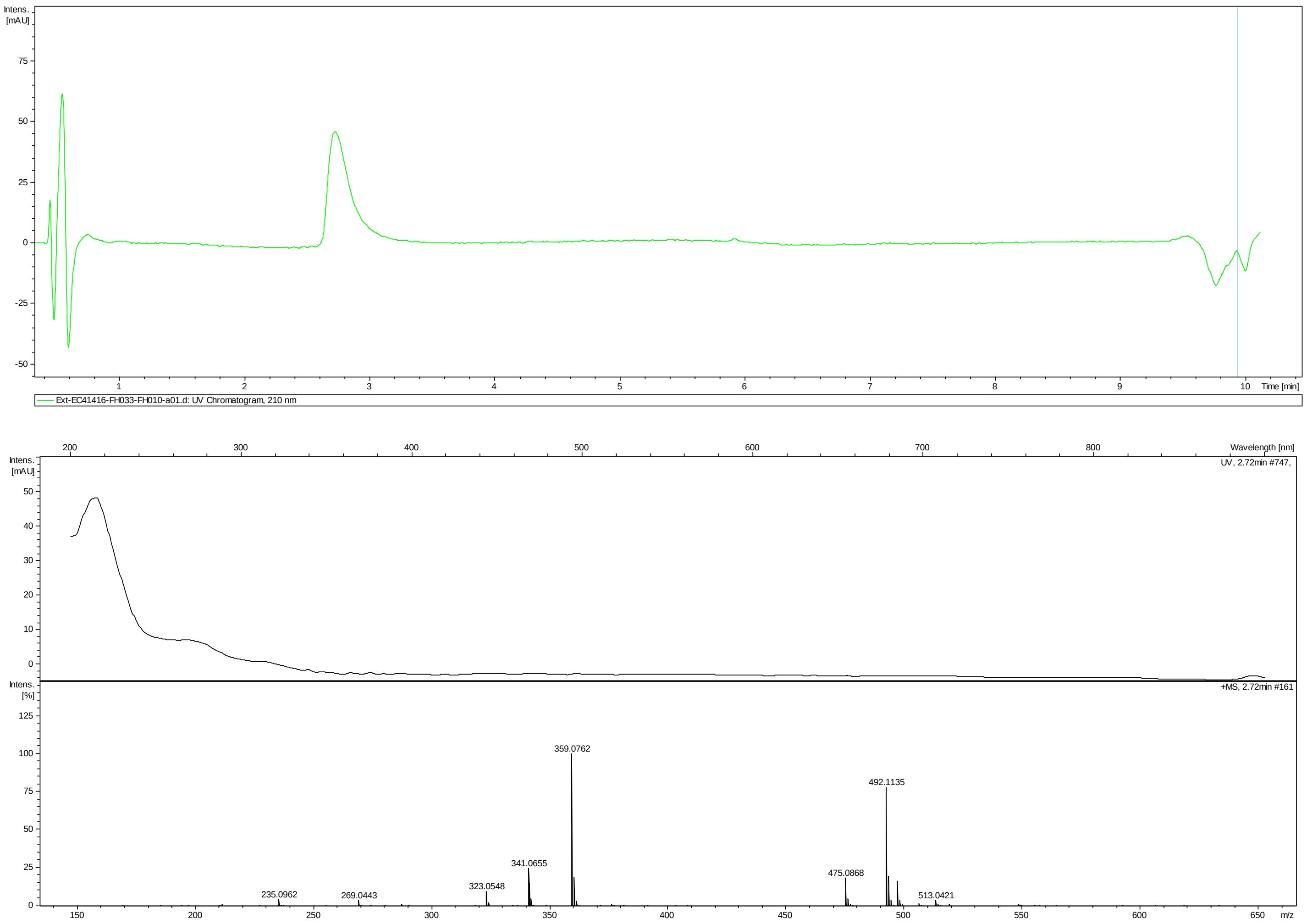

Figure S1. a) LC-UV-HRMS chromatogram (UV 210 nm). b) UV spectrum of 8-Hydroxyfumarprotocetraric acid. c) HRESIMS-MS(+)-TOF spectra of 8-Hydroxyfumarprotocetraric acid.

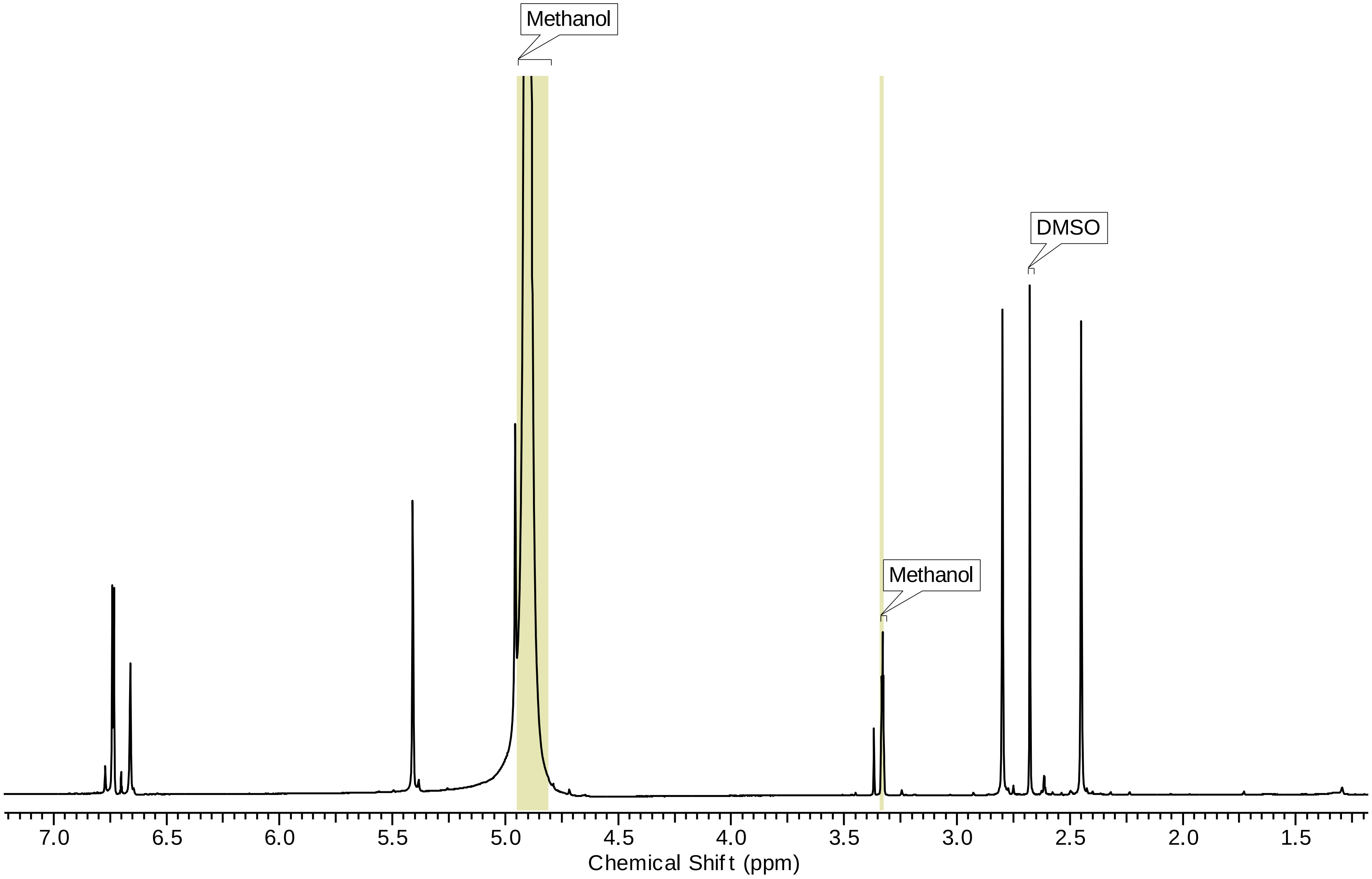

Figure S2. ^{1}H spectra of 8-Hydroxyfumarprotocetraric acid.

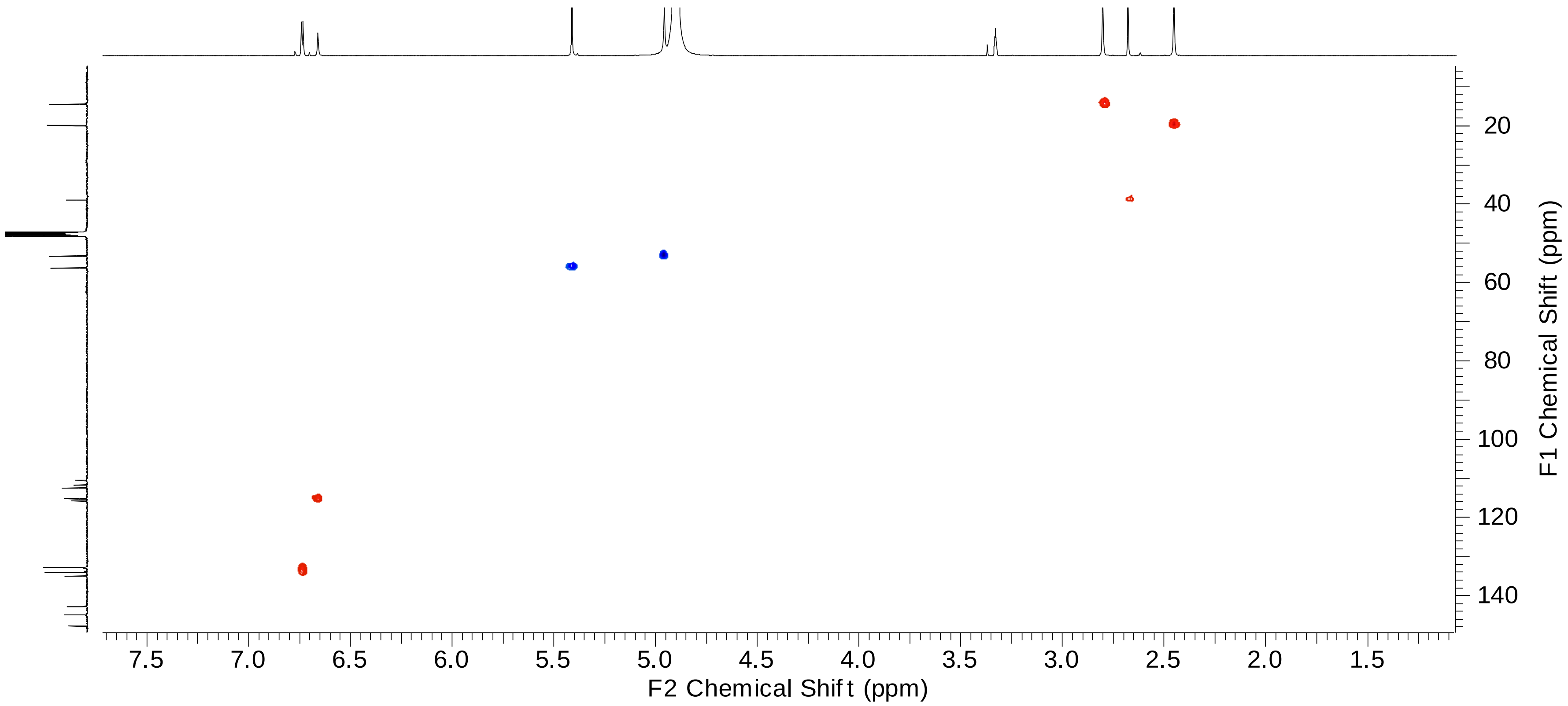

Figure S3. HSQC spectra of 8-Hydroxyfumarprotocetraric acid.

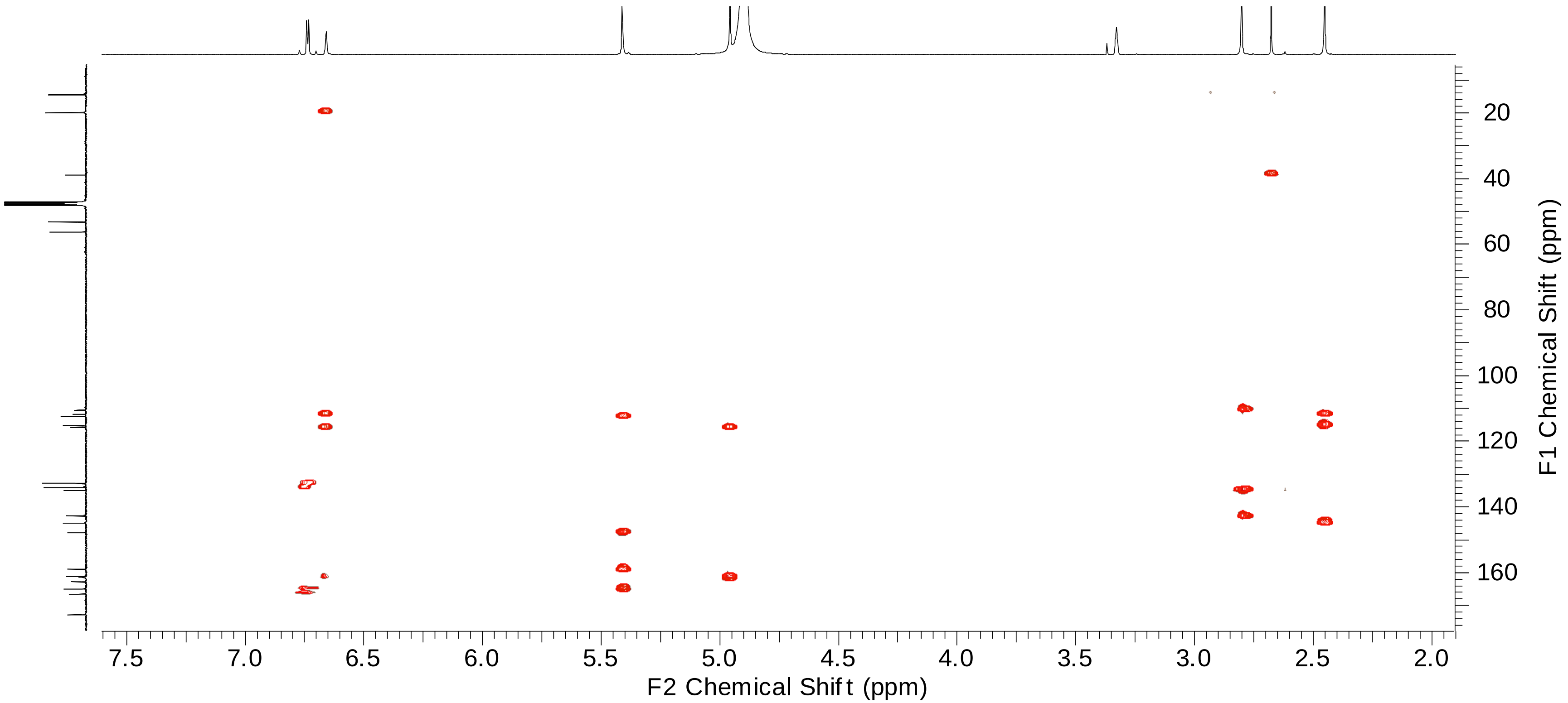

Figure S4. HMBC spectra of 8-Hydroxyfumarprotocetraric acid.

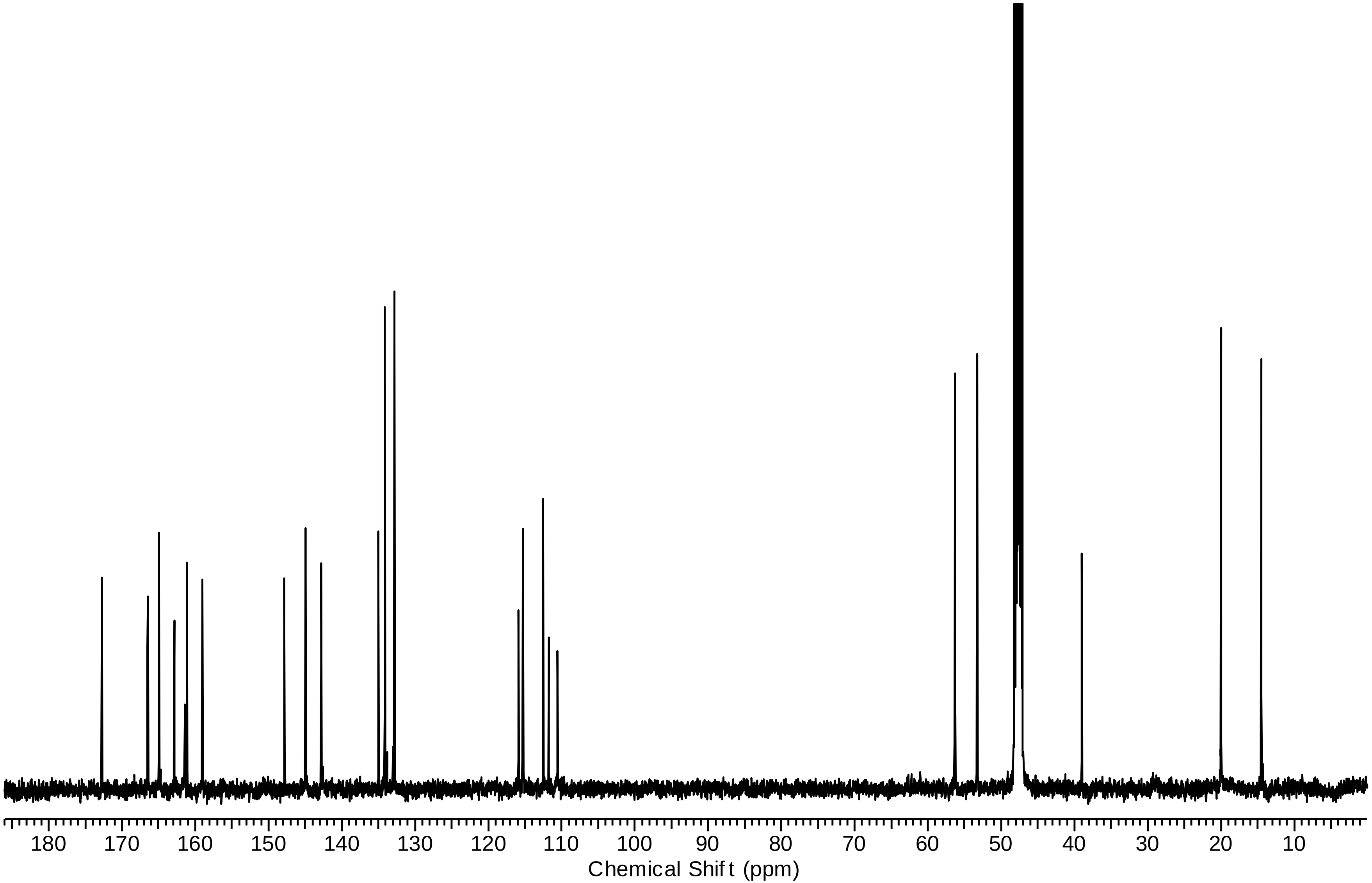

Figure S5. ^{13}C spectra of 8-Hydroxyfumarprotocetraric acid.

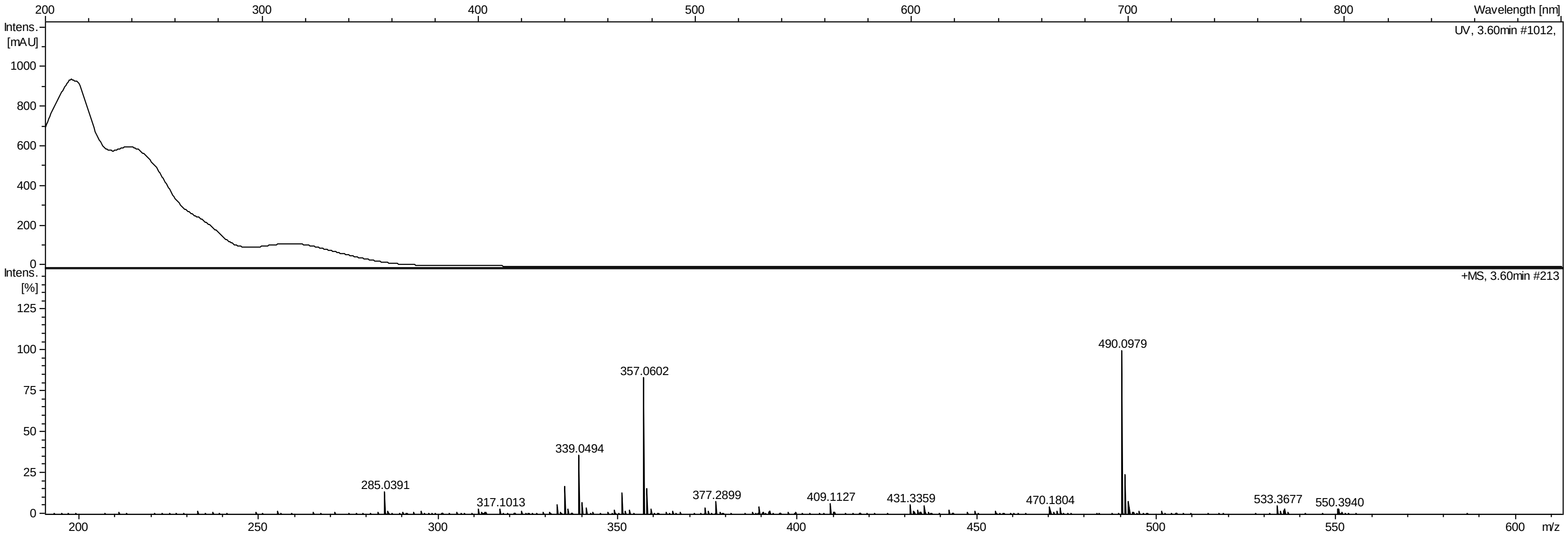

Figure S6. a) UV spectrum of fumarprotocetraric acid. b) HRESIMS-MS(+)-TOF spectra of fumarprotocetraric acid.

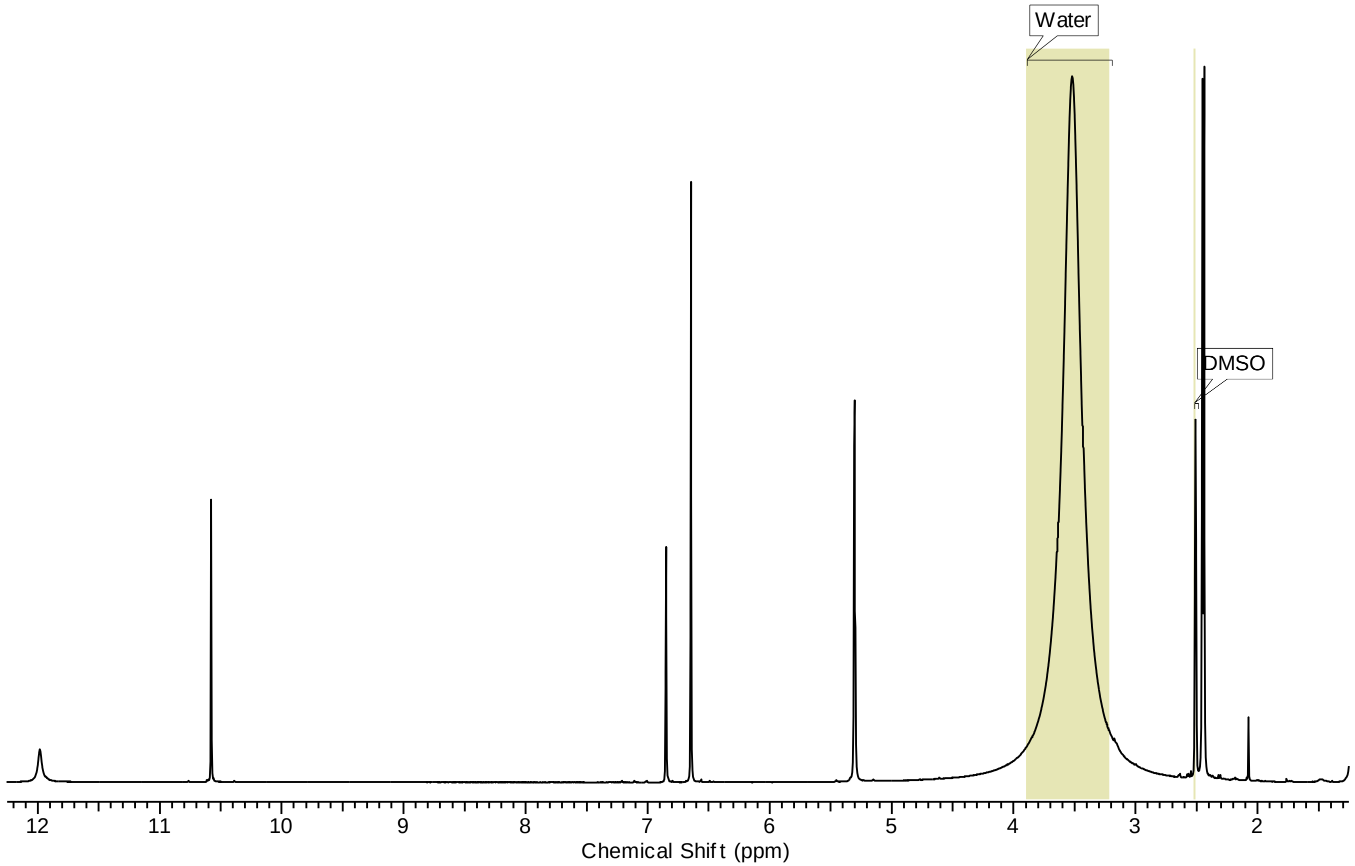

Figure S7. ^{1}H spectra of fumaroprotocetraric acid.

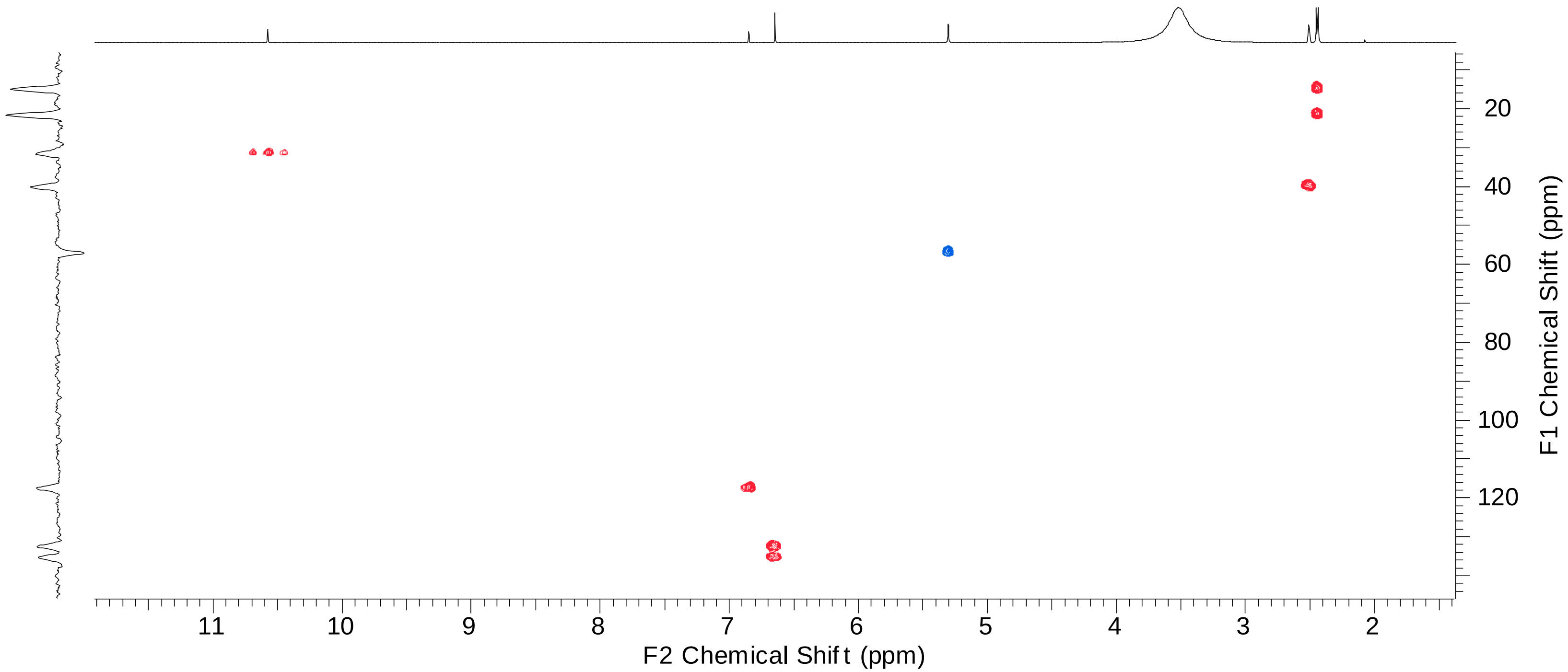

Figure S8. HSQC spectra of fumaroprotocetraric acid.

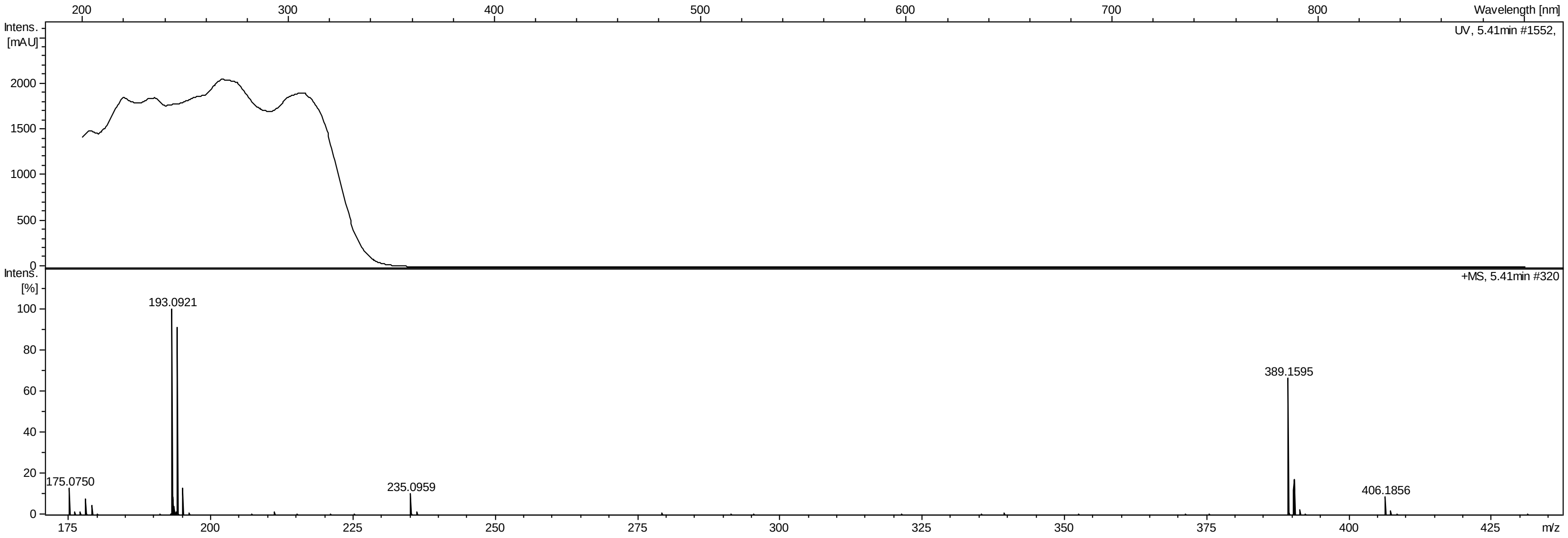

Figure S9. a) UV spectrum of divaricatic acid. b) HRESIMS-MS(+)-TOF spectra of divaricatic acid.

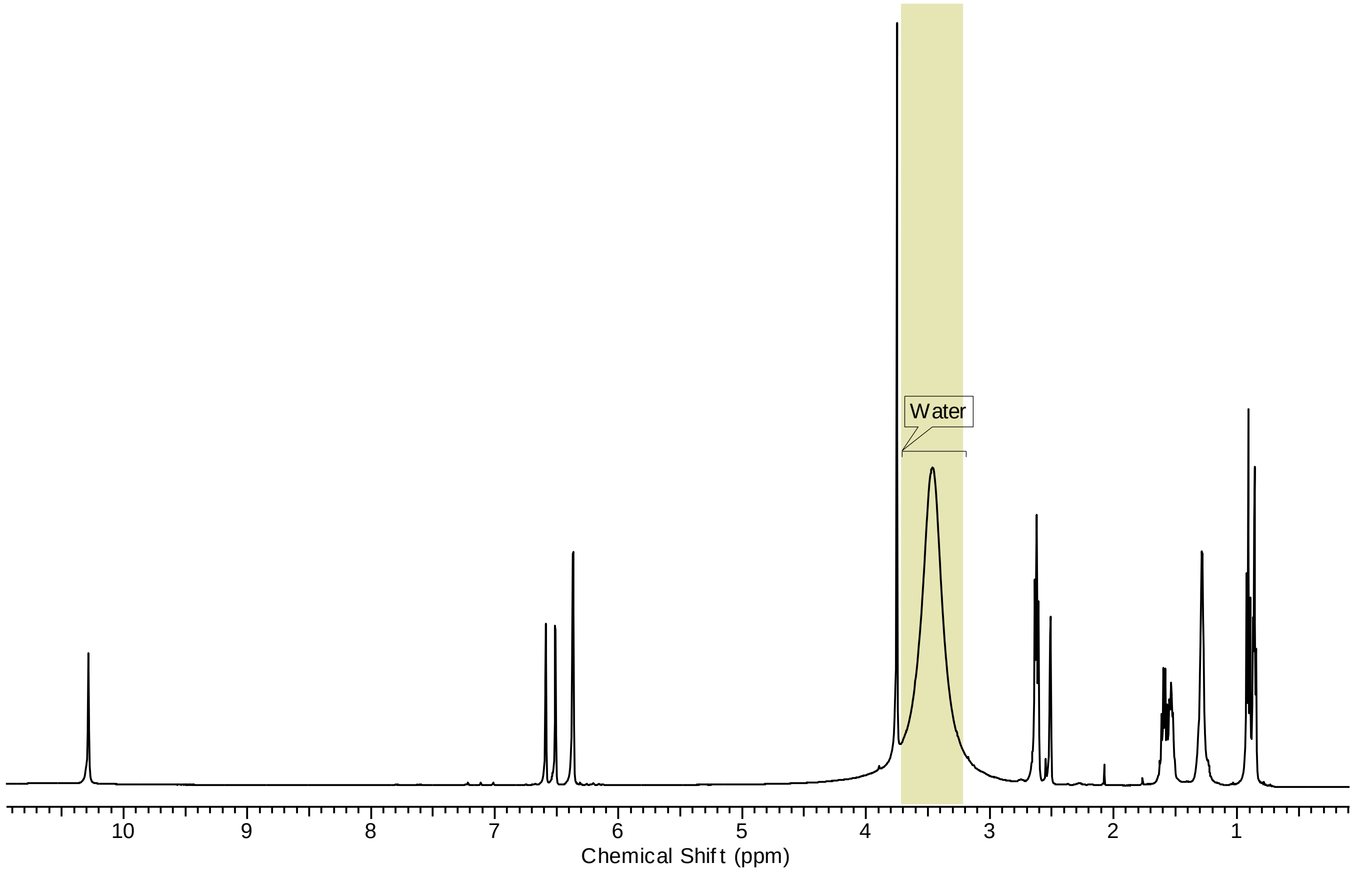

Figure S10. ^{1}H spectra of divaricatic acid.

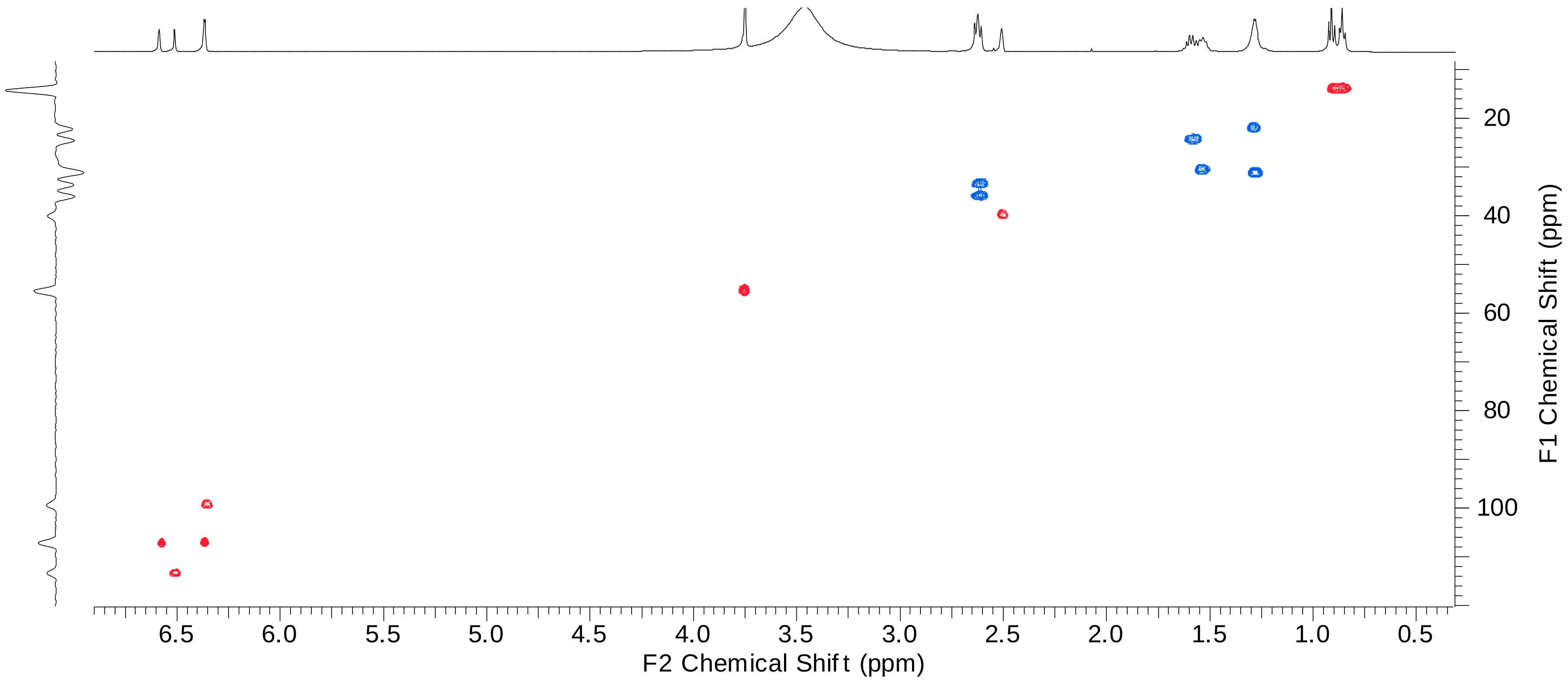

Figure S11. HSQC spectra of divaricatic acid.

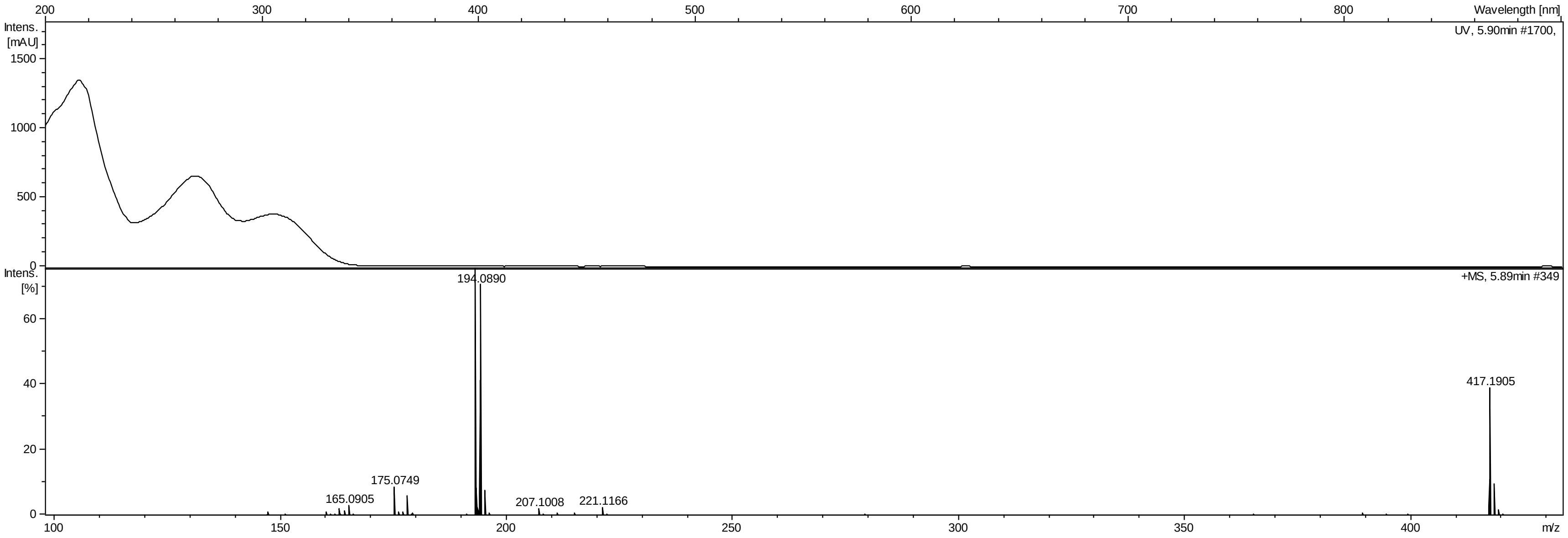

Figure S12. a) UV spectrum of stenosporic acid. b) HRESIMS-MS(+)-TOF spectra of stenosporic acid.

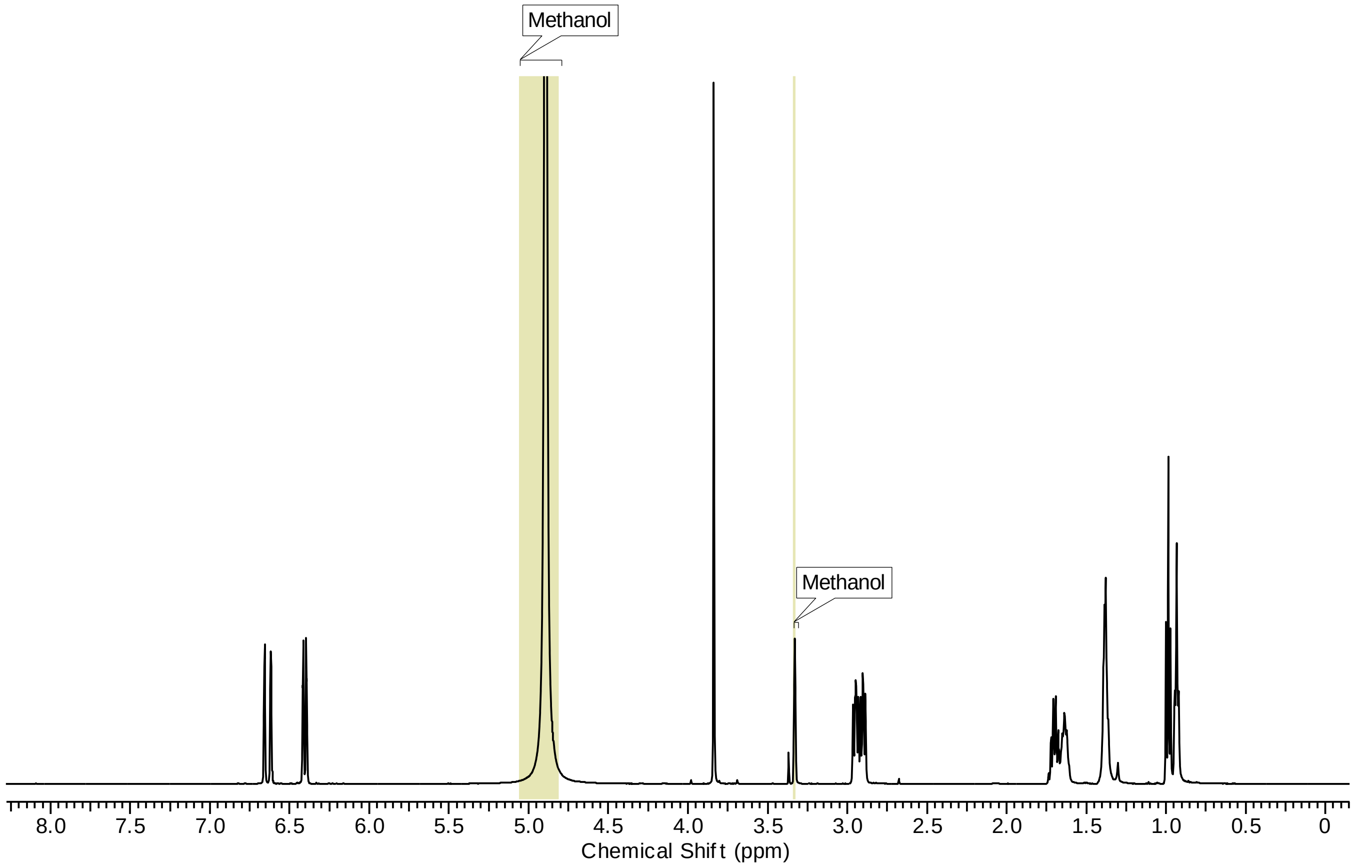

Figure S13. ^{1}H spectra of stenosporic acid.

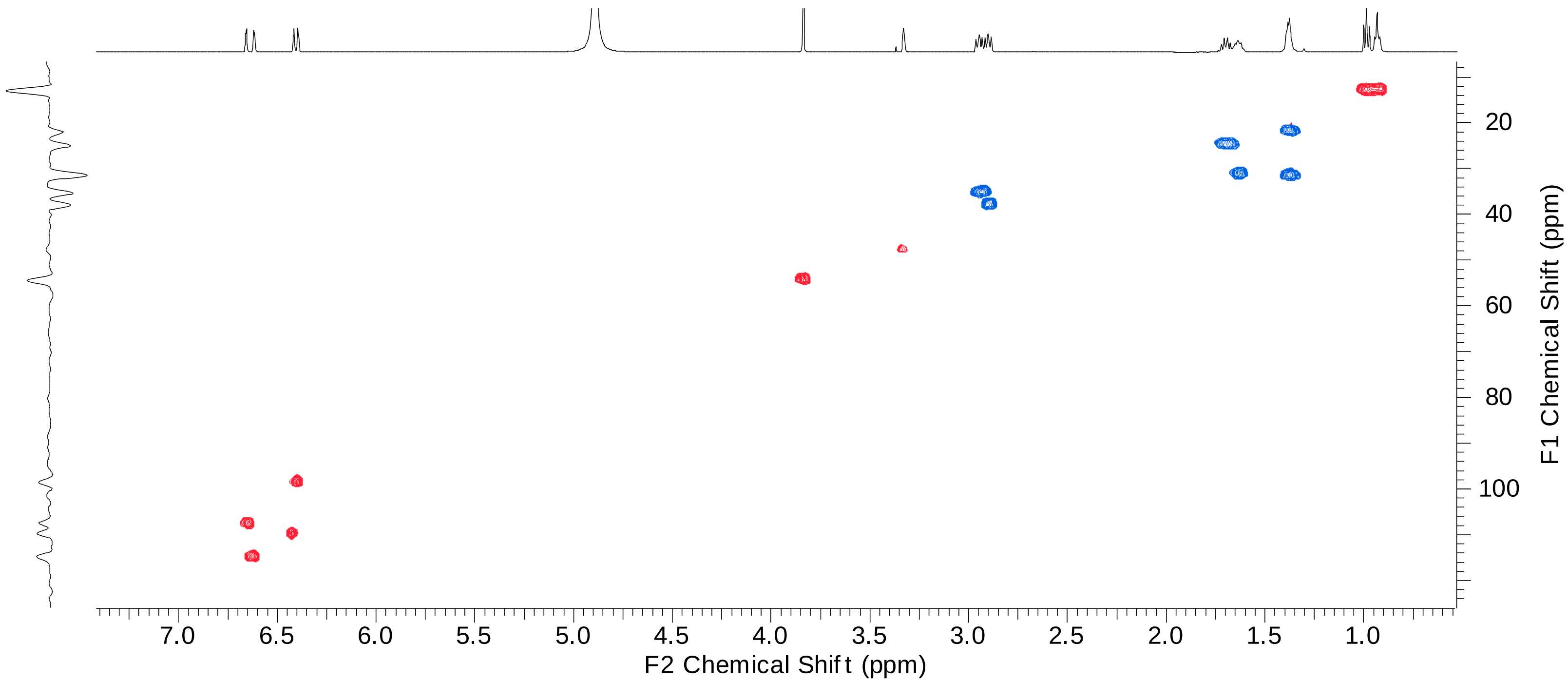

Figure S14. HSQC spectra of stenosporic acid.

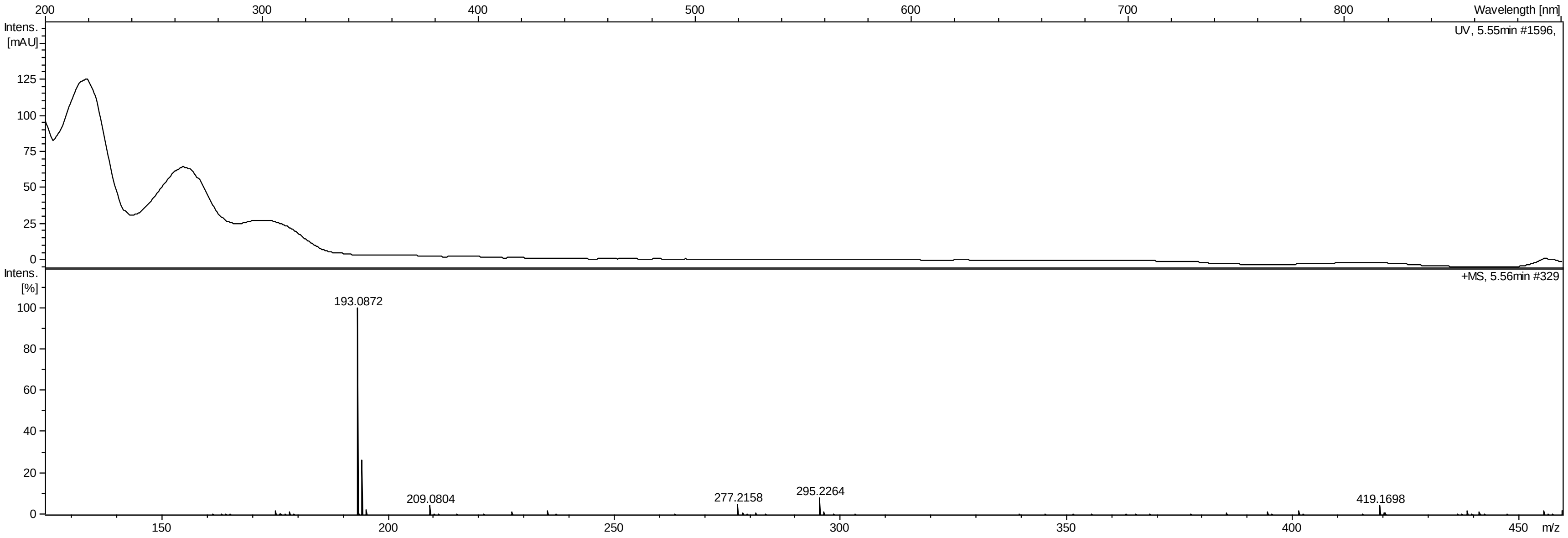

Figure S15. a) UV spectrum of sekikaic acid. b) HRESIMS-MS(+)-TOF spectra of sekikaic acid.

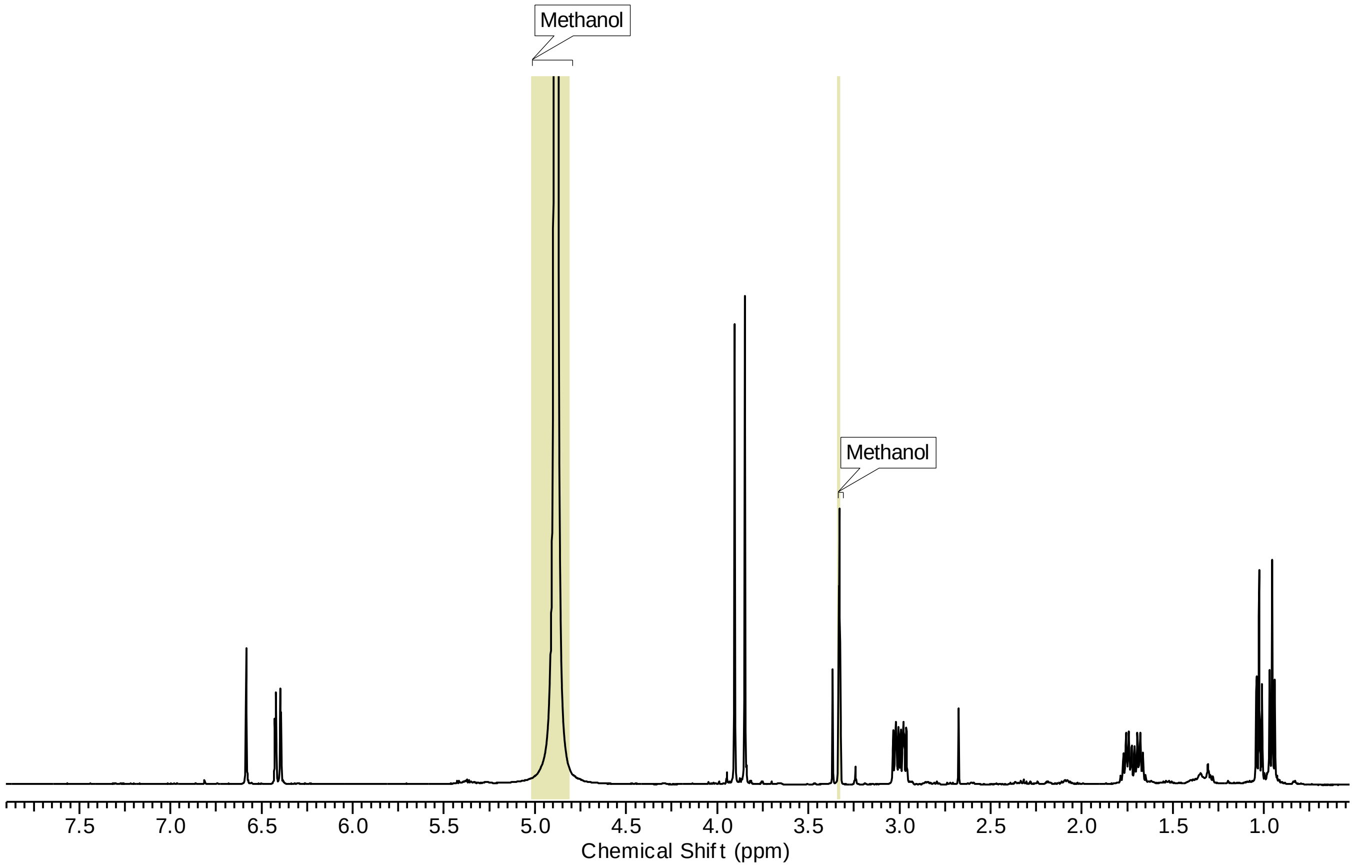

Figure S16. ^{1}H spectra of sekikaic acid.

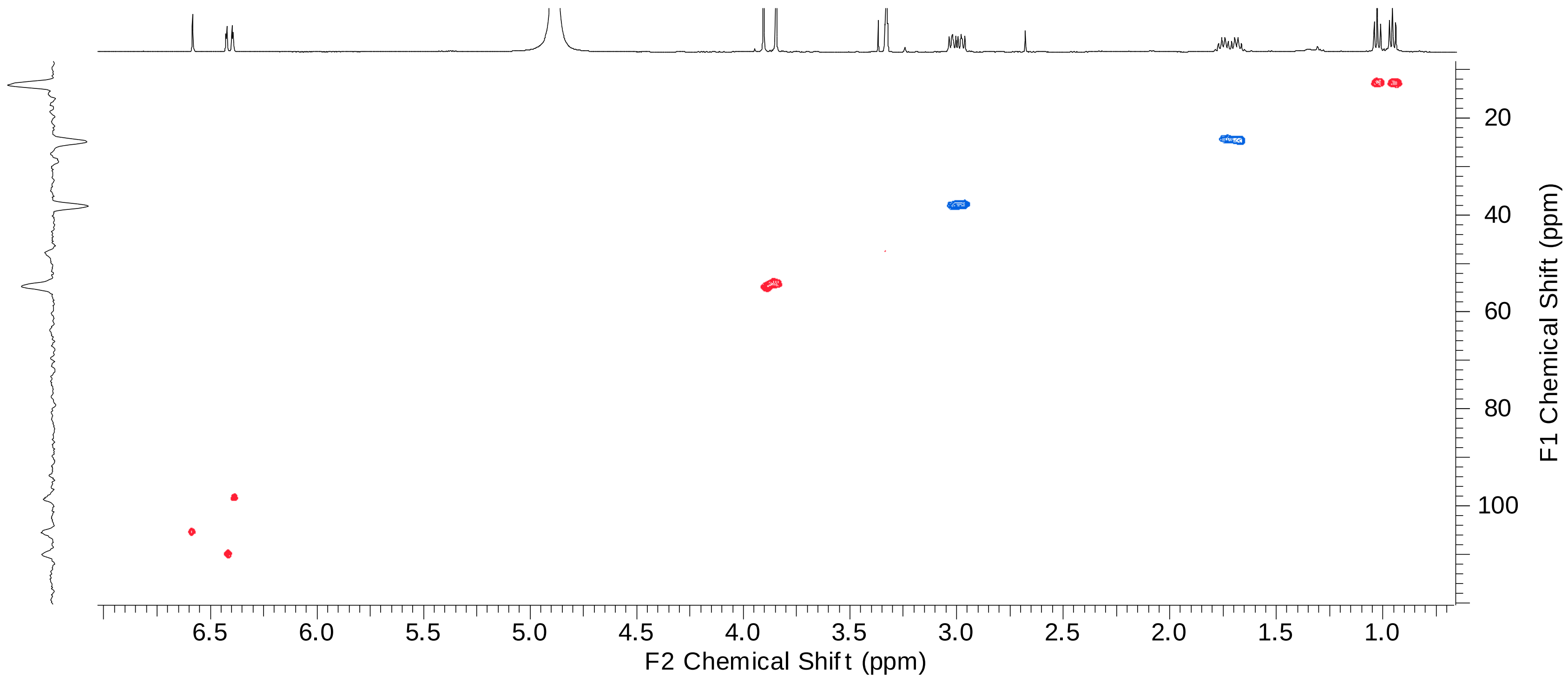

Figure S17. HSQC spectra of sekikaic acid.

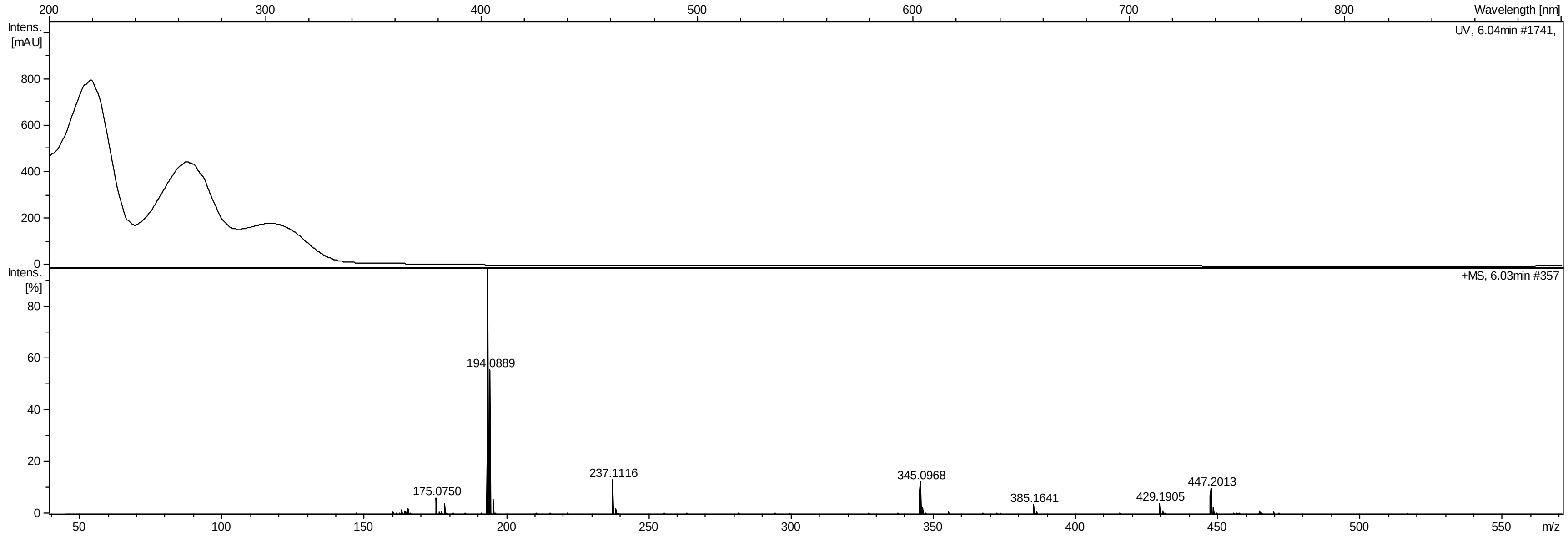

Figure S18. a) UV spectrum of 4′-O-Methylpaludosic acid. b) HRESIMS-MS(+)-TOF spectra of 4′-O-Methylpaludosic acid.

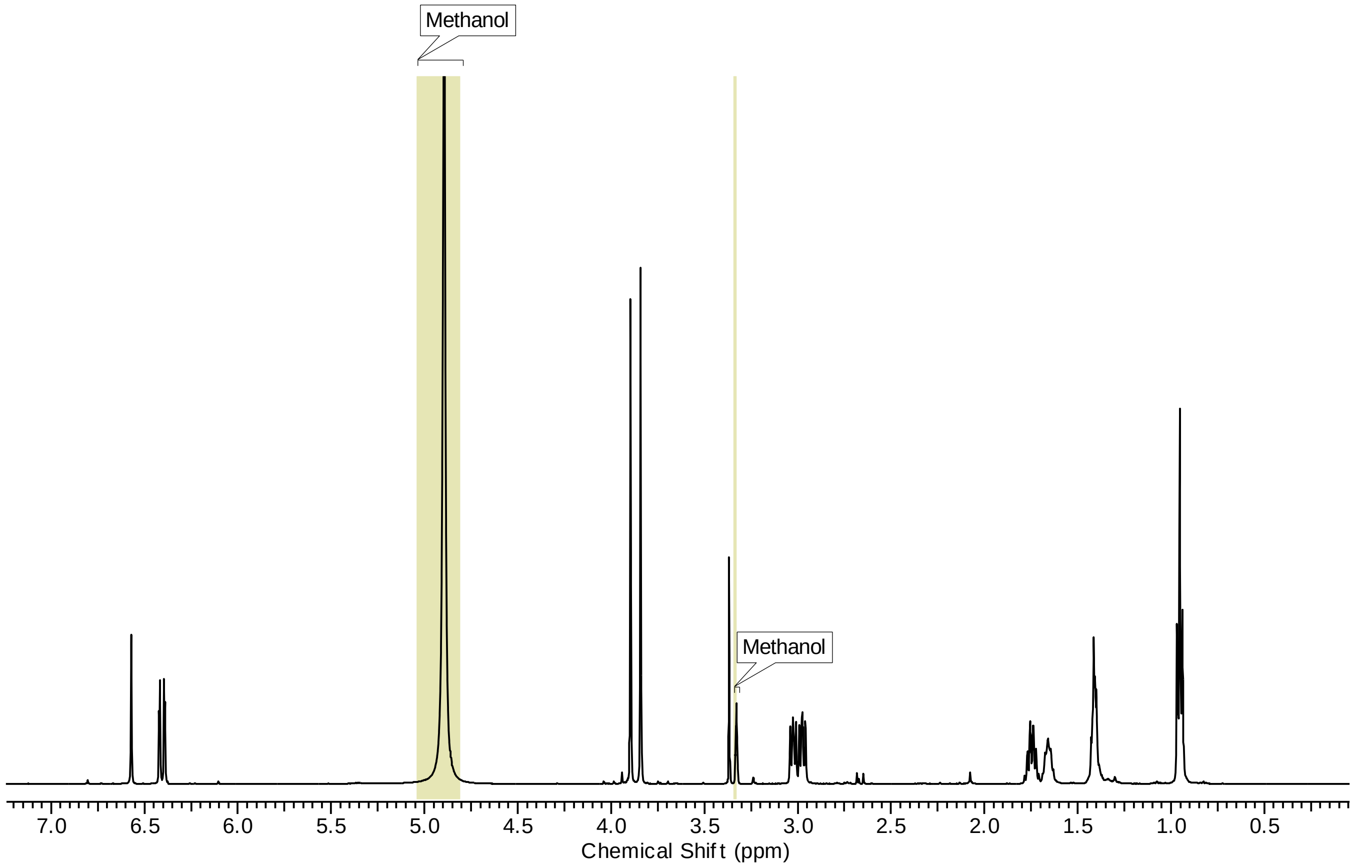

Figure S19. ^{1}H spectra of 4′-O-Methylpaludosic acid.

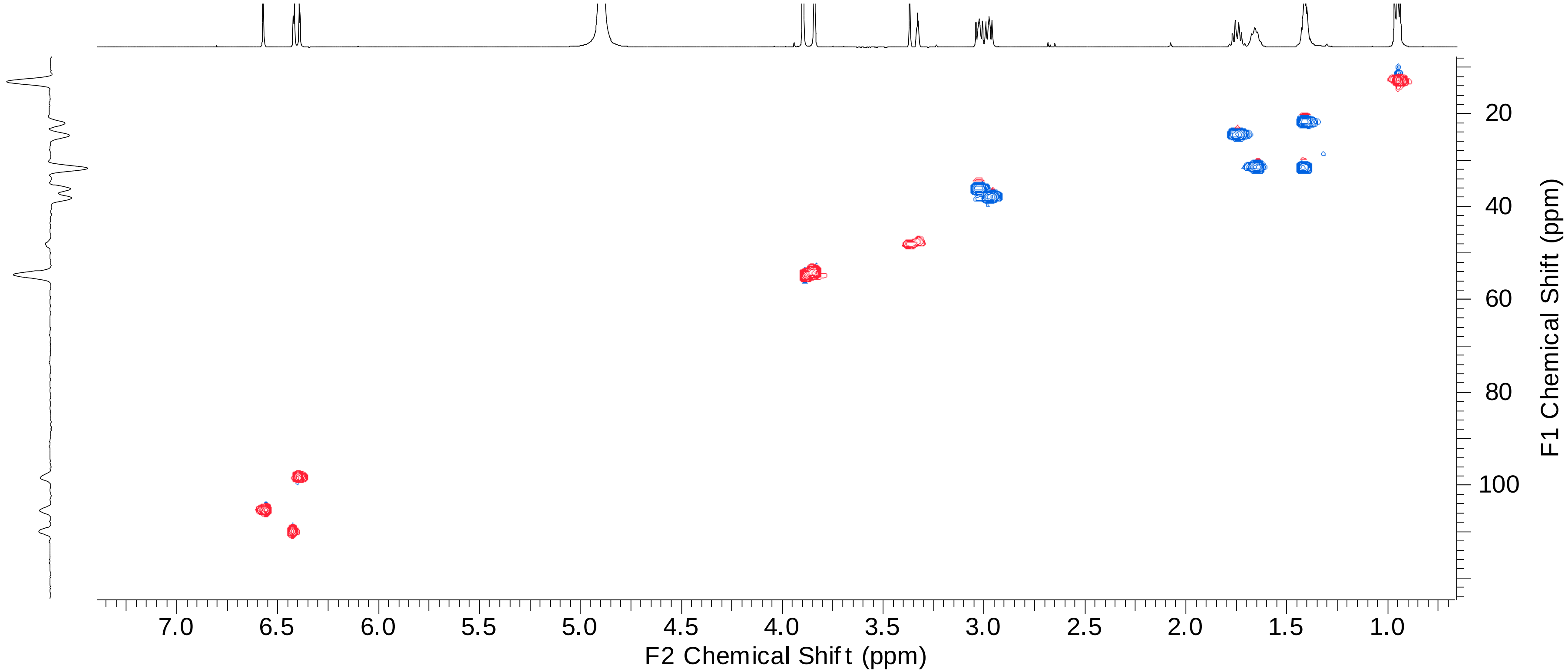

Figure S20. HSQC spectra of 4′-O-Methylpaludosic acid.

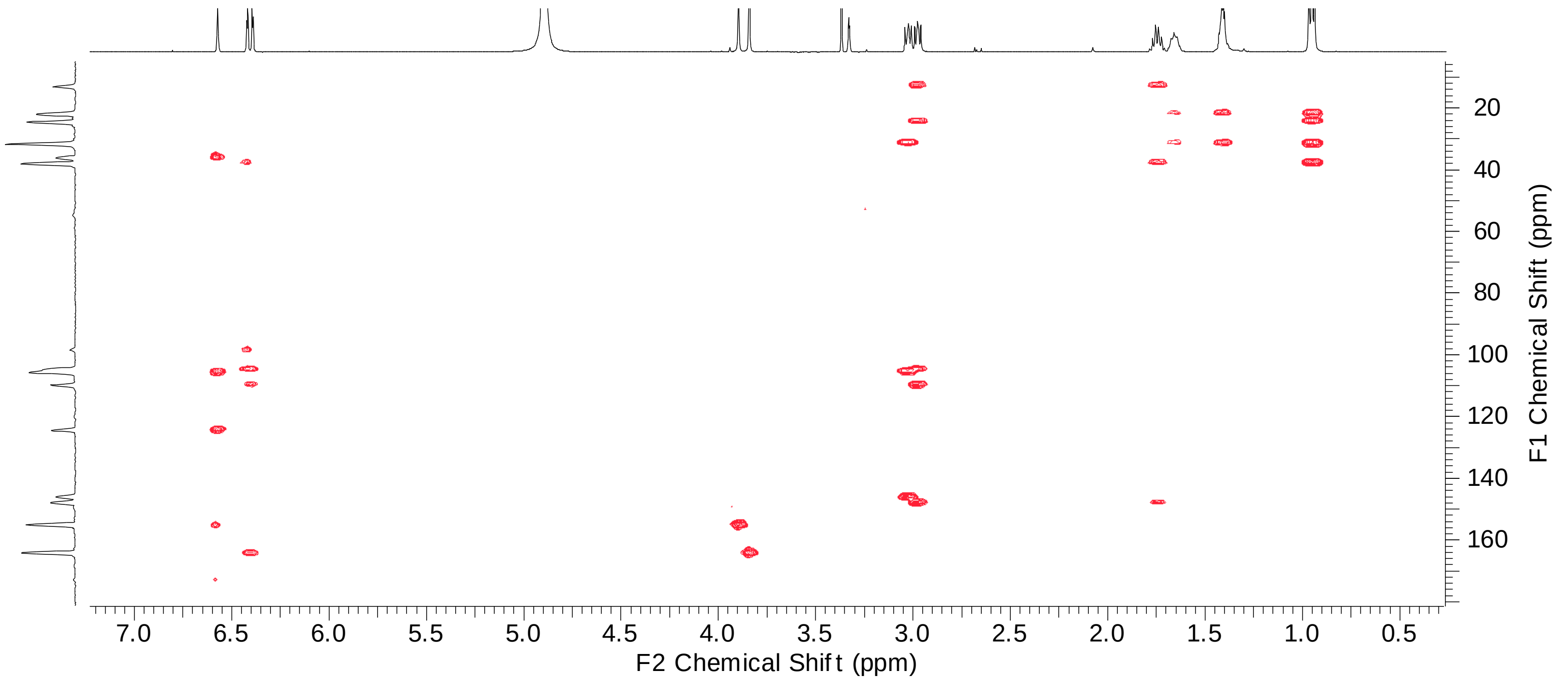

Figure S21. HMBC spectra of 4′-O-Methylpaludosic acid.

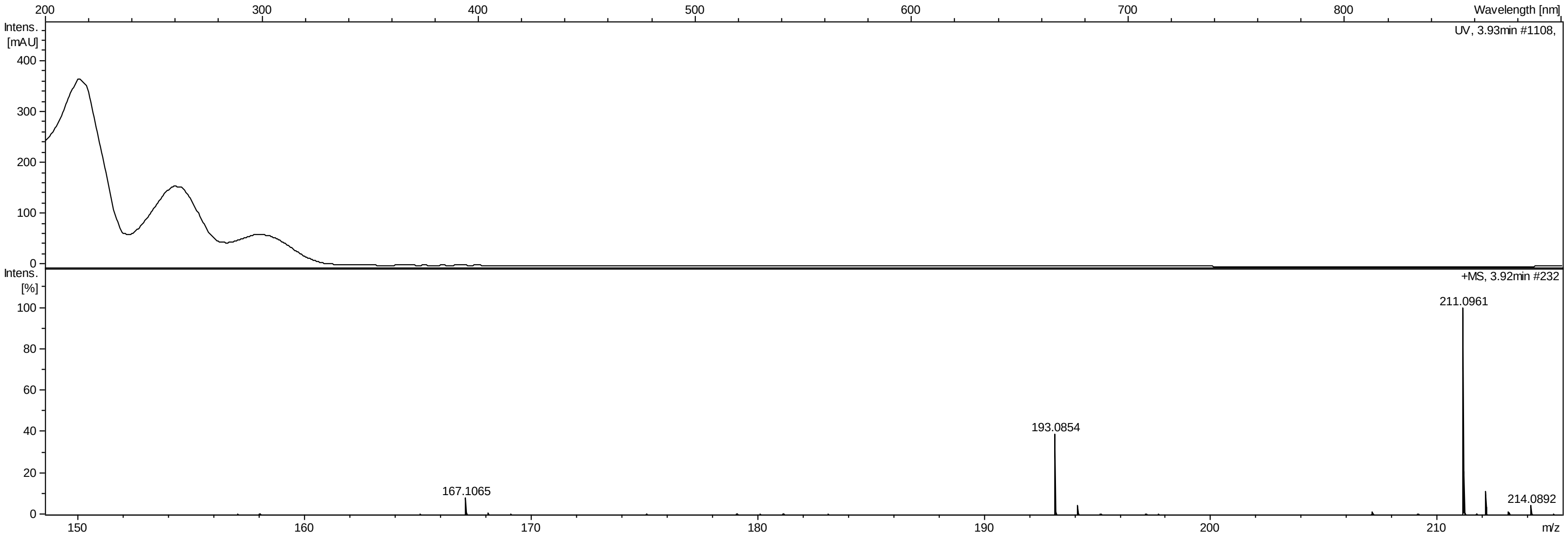

Figure S22. a) UV spectrum of ethyl everninate. b) HRESIMS-MS(+)-TOF spectra of ethyl everninate.

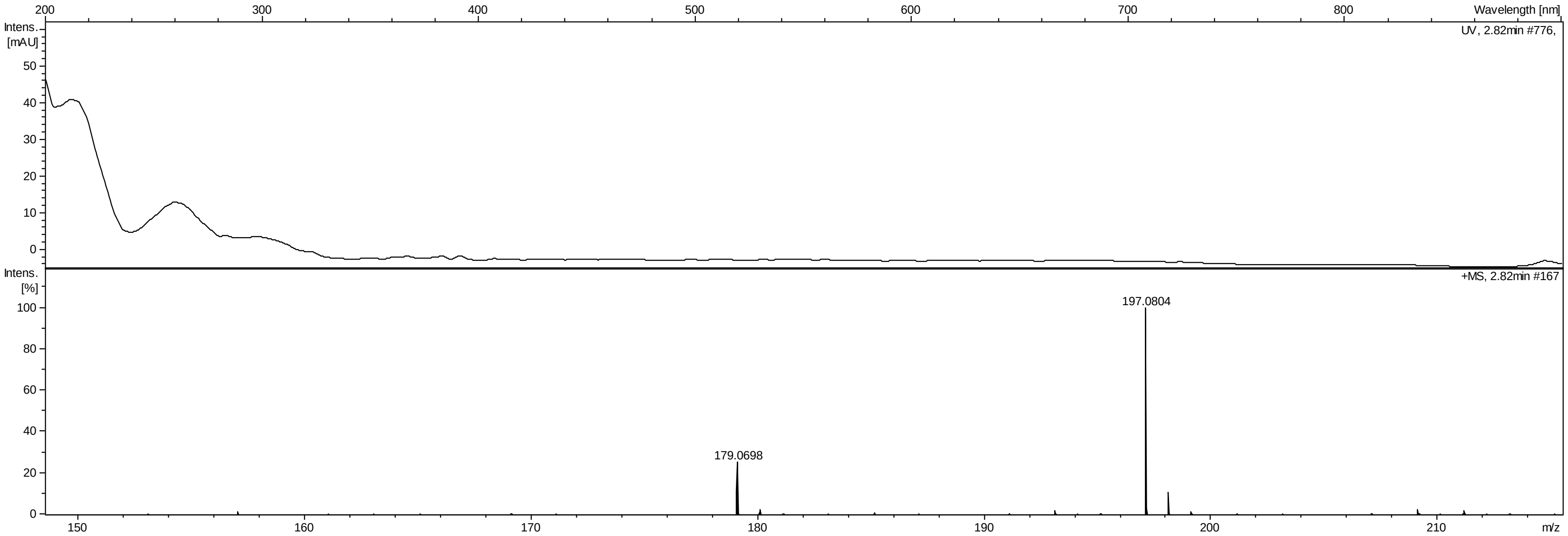

Figure S23. a) UV spectrum of divaric acid. b) HRESIMS-MS(+)-TOF spectra of divaric acid.

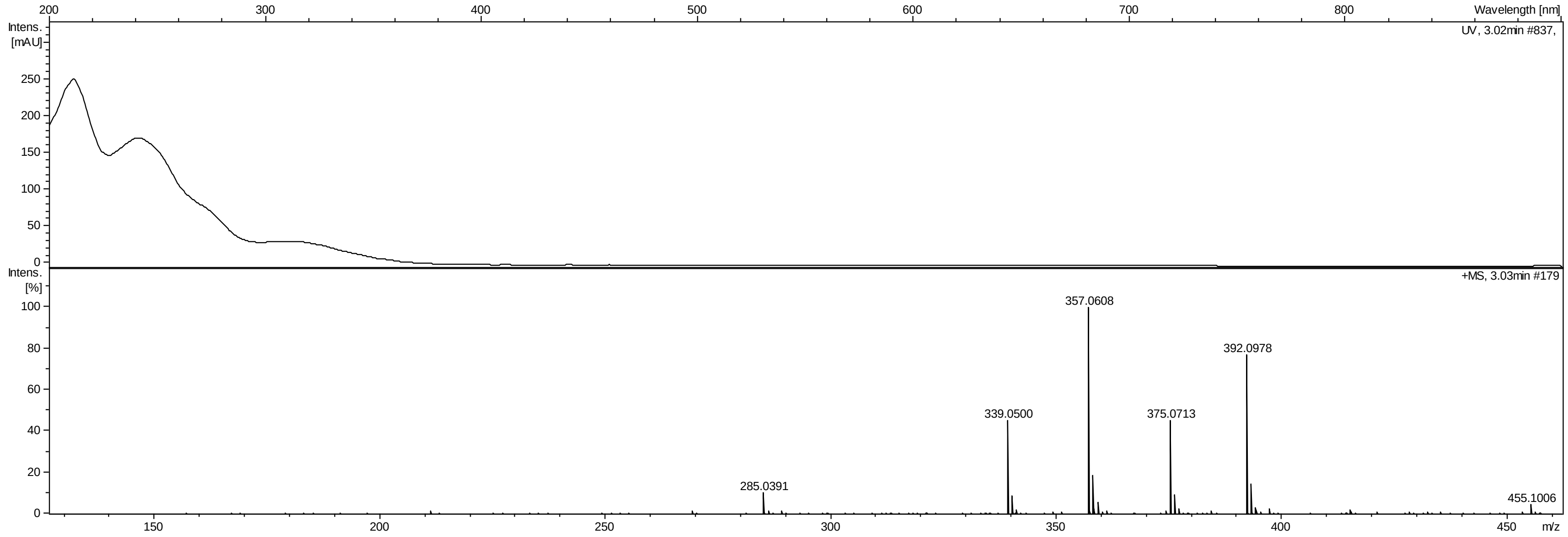

Figure S24. a) UV spectrum of protocetraric acid. b) HRESIMS-MS(+)-TOF spectra of protocetraric acid.

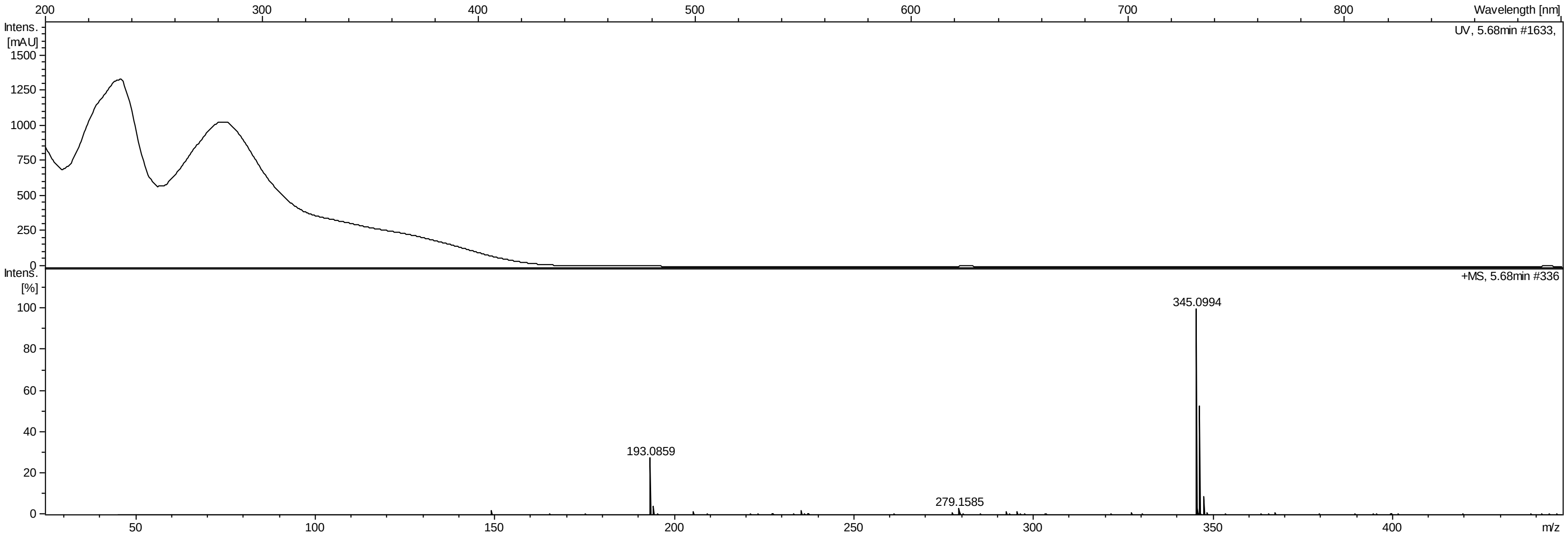

Figure S25. a) UV spectrum of usnic acid. b) HRESIMS-MS(+)-TOF spectra of usnic acid.

Zymoseptoria tritici Absorbance Based Assay

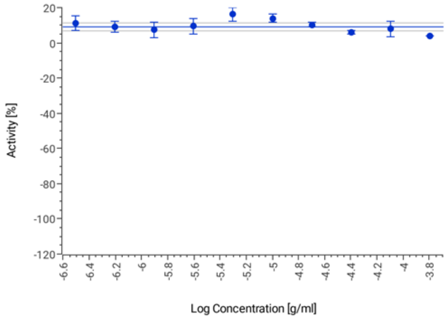

Compound ID	qAC50 [µg/mL]	Lower 95% CL [µg/mL]	Upper 95% CL [µg/mL]	R^2
8-hydroxyfumarprotocetraric acid	> 160	-	-	-

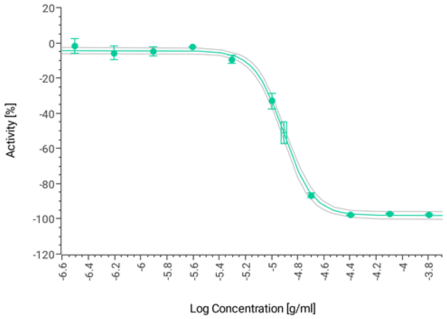

Compound ID	qAC50 [µg/mL]	Lower 95% CL [µg/mL]	Upper 95% CL [µg/mL]	R^2
Divaricatic acid	12.40	11.81	13.01	0.99

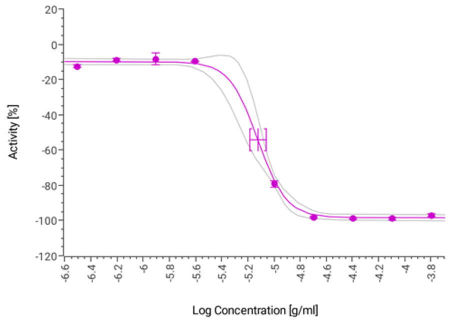

Compound ID	qAC50 [µg/mL]	Lower 95% CL [µg/mL]	Upper 95% CL [µg/mL]	R^2
Stenosporic acid	7.53	6.52	8.70	0.99

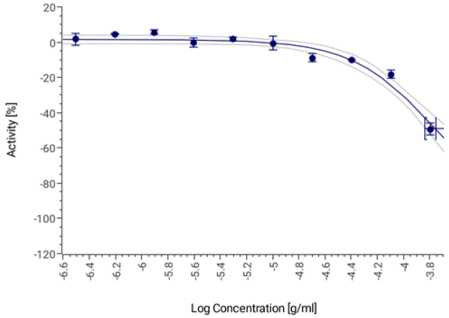

Compound ID	qAC50 [µg/mL]	Lower 95% CL [µg/mL]	Upper 95% CL [µg/mL]	R^2
Sekikaic acid	> 160	-	-	0.90

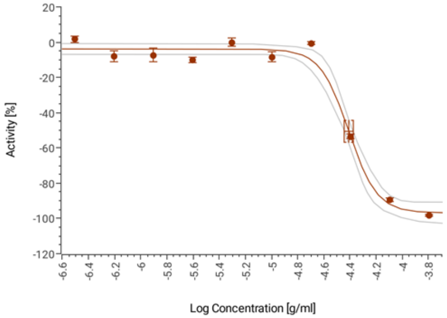

Compound ID	qAC50 [µg/mL]	Lower 95% CL [µg/mL]	Upper 95% CL [µg/mL]	R^2
4'-O-methylpaludosic acid	38.87	35.78	42.23	0.98

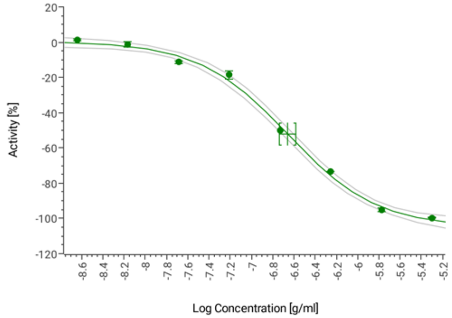

Compound ID	qAC50 [g/ml]	Lower 95% CL [g/ml]	Upper 95% CL [g/ml]	R^2
Amphotericin B	0.22	0.18	0.26	0.99

Figure S26. Dose Response Curves, EC50 values and their corresponding 95% confidence limits for compounds 1-5 and Amphotericin B obtained from *Zymoseptoria tritici* absorbance-based assay.

Zymoseptoria tritici Fluorescence Based Assay

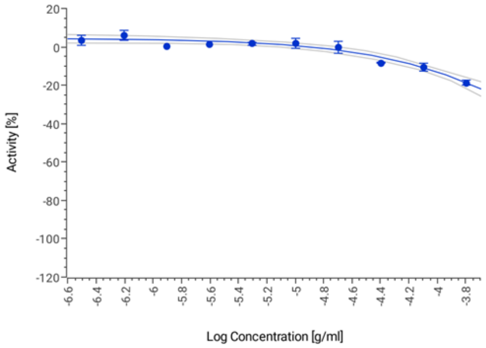

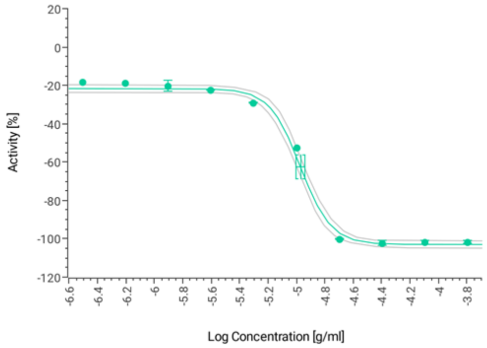

Compound ID	qAC50 [µg/mL]	Lower 95% CL [µg/mL]	Upper 95% CL [µg/mL]	R^2
Divaricatic acid	10.67	9.98	11.41	0.99

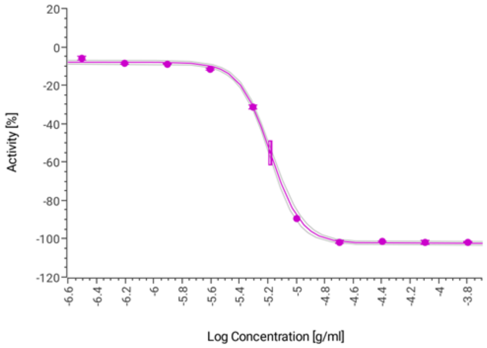

Compound ID	qAC50 [µg/mL]	Lower 95% CL [µg/mL]	Upper 95% CL [µg/mL]	R^2
Stenosporic acid	6.59	6.39	6.79	0.99

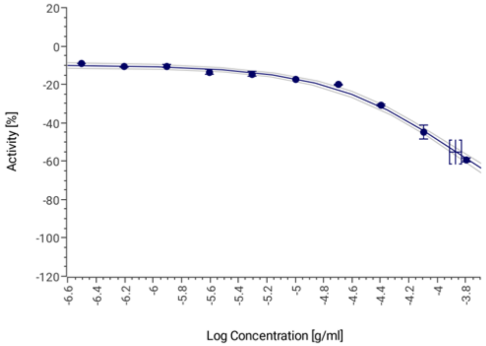

Compound ID	qAC50 [µg/mL]	Lower 95% CL [µg/mL]	Upper 95% CL [µg/mL]	R^2
Sekikaic acid	134.6	122.7	147.6	0.99

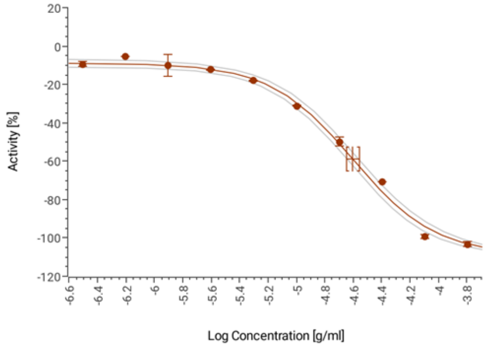

Compound ID	qAC50 [µg/mL]	Lower 95% CL [µg/mL]	Upper 95% CL [µg/mL]	R^2
4'-O-methylpaludosic acid	24.88	22.33	27.73	0.99

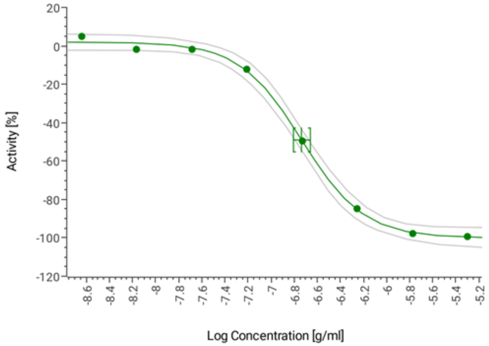

Compound ID	qAC50 [g/ml]	Lower 95% CL [g/ml]	Upper 95% CL [g/ml]	R^2
Amphotericin B	0.19	0.16	0.22	0.97

Figure S27. Dose Response Curves, EC50 values and their corresponding 95% confidence limits for compounds 1-5 and Amphotericin B obtained from *Zymoseptoria tritici* fluorescence-based assay.

Botrytis cinerea Absorbance Based Assay

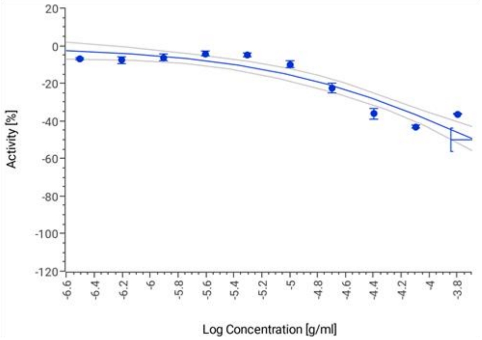

Compound ID	qAC50 [µg/mL]	Lower 95% CL [µg/mL]	Upper 95% CL [µg/mL]	R^2
8-hydroxyfumarprotocetraric acid	> 160	-	-	0.86

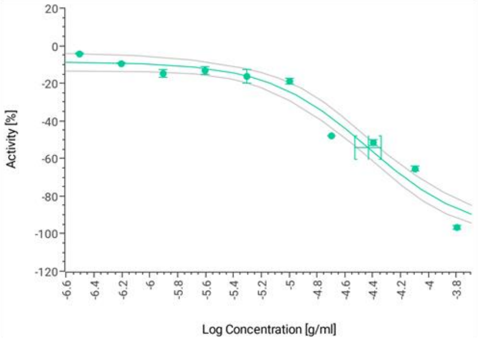

Compound ID	qAC50 [µg/mL]	Lower 95% CL [µg/mL]	Upper 95% CL [µg/mL]	R^2
Divaricatic acid	36.87	29.72	45.73	0.94

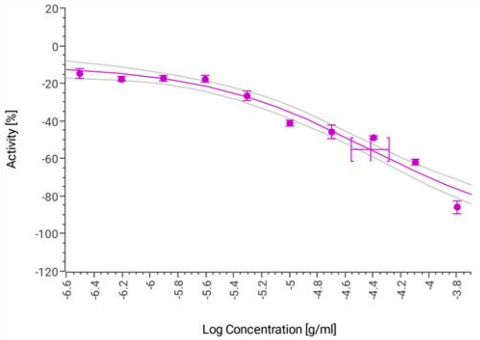

Compound ID	qAC50 [µg/mL]	Lower 95% CL [µg/mL]	Upper 95% CL [µg/mL]	R^2
Stenosporic acid	38.03	27.77	52.09	0.94

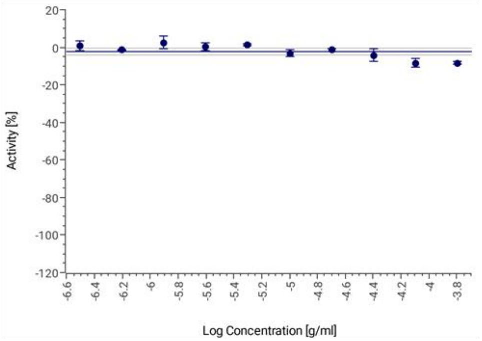

Compound ID	qAC50 [µg/mL]	Lower 95% CL [µg/mL]	Upper 95% CL [µg/mL]	R^2
Sekikaic acid	> 160	-	-	-

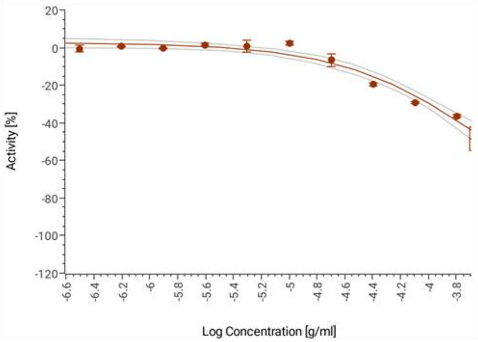

Compound ID	qAC50 [µg/mL]	Lower 95% CL [µg/mL]	Upper 95% CL [µg/mL]	R^2
4'-O-methylpaludosic acid	> 160	-	-	0.93

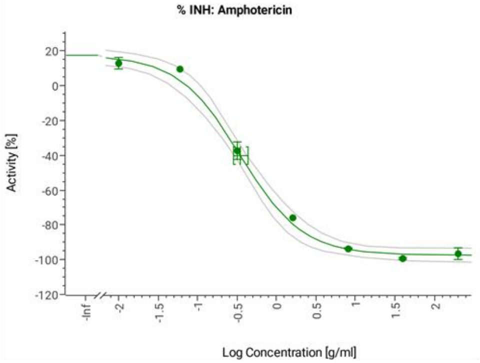

Compound ID	qAC50 [g/ml]	Lower 95% CL [g/ml]	Upper 95% CL [g/ml]	R^2
Amphotericin B	0.35	0.28	0.43	0.99

Figure S28. Dose Response Curves, EC50 values and their corresponding 95% confidence limits for compounds 1-5 and Amphotericin B obtained from *B. cinerea* absorbance-based assay.

Botrytis cinerea Fluorescence Based Assay

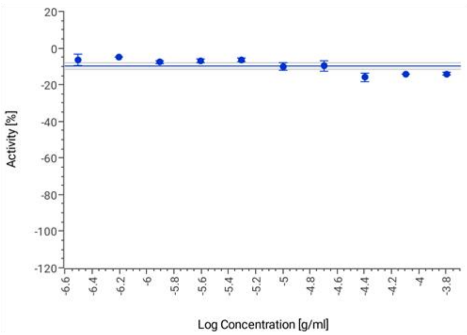

Compound ID	qAC50 [µg/mL]	Lower 95% CL [µg/mL]	Upper 95% CL [µg/mL]	R^2
8-hydroxyfumarprotocetraric acid	> 160	-	-	-

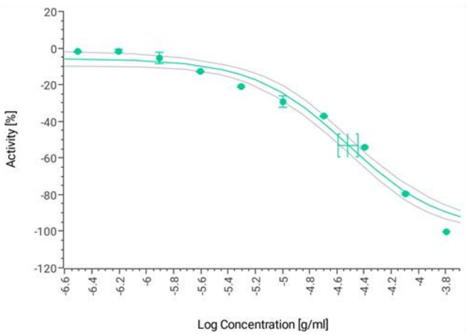

Compound ID	qAC50 [µg/mL]	Lower 95% CL [µg/mL]	Upper 95% CL [µg/mL]	R^2
Divaricatic acid	30.29	25.57	35.88	0.97

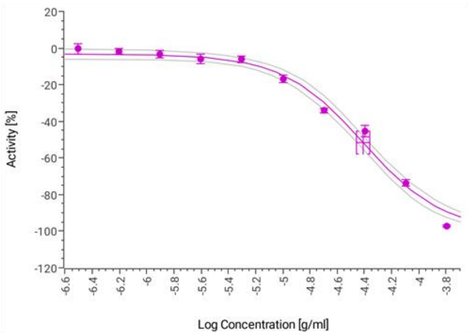

Compound ID	qAC50 [µg/mL]	Lower 95% CL [µg/mL]	Upper 95% CL [µg/mL]	R^2
Stenosporic acid	38.94	34.78	43.59	0.98

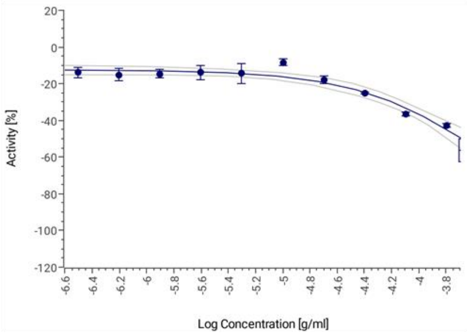

Compound ID	qAC50 [µg/mL]	Lower 95% CL [µg/mL]	Upper 95% CL [µg/mL]	R^2
Sekikaic acid	> 160	-	-	0.87

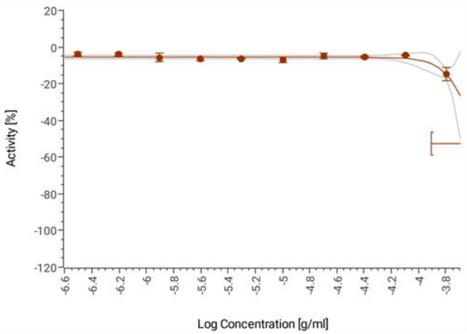

Compound ID	qAC50 [µg/mL]	Lower 95% CL [µg/mL]	Upper 95% CL [µg/mL]	R^2
4'-O-methylpaludosic acid	> 160	-	-	0.63

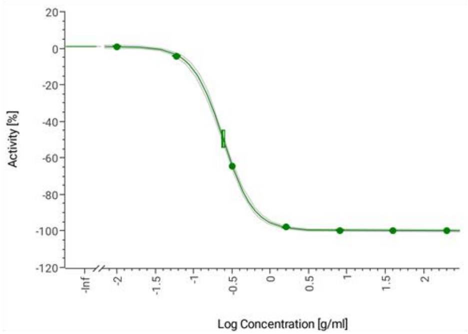

Compound ID	qAC50 [g/ml]	Lower 95% CL [g/ml]	Upper 95% CL [g/ml]	R^2
Amphotericin B	0.24	0.23	0.25	0.99

Figure S29. Dose Response Curves, EC50 values and their corresponding 95% confidence limits for compounds 1-5 and Amphotericin B obtained from *B. cinerea* fluorescence-based assay.

Colletotrichum acutatum Absorbance Based Assay

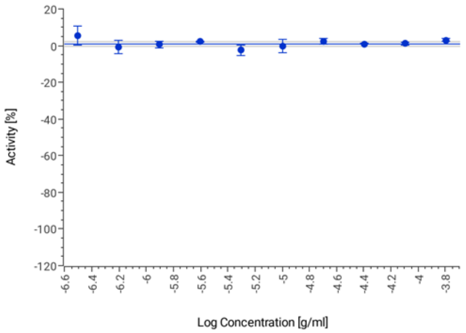

Compound ID	qAC50 [µg/mL]	Lower 95% CL [µg/mL]	Upper 95% CL [µg/mL]	R^2
8-hydroxyfumarprotocetraric acid	> 160	-	-	-

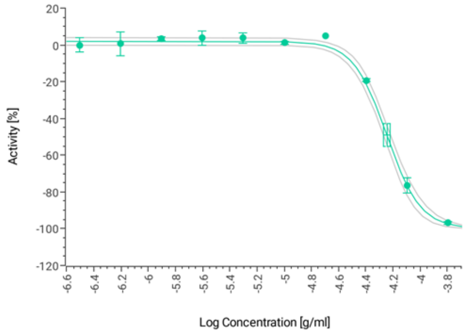

Compound ID	qAC50 [µg/mL]	Lower 95% CL [µg/mL]	Upper 95% CL [µg/mL]	R^2
Divaricatic acid	56.84	53.58	60.29	0.99

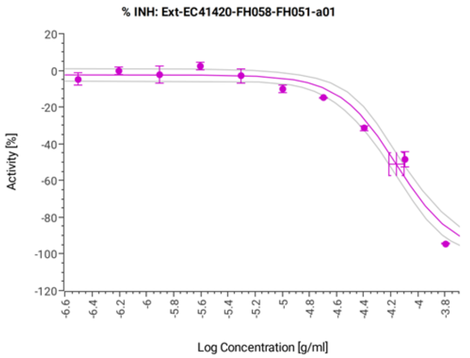

Compound ID	qAC50 [µg/mL]	Lower 95% CL [µg/mL]	Upper 95% CL [µg/mL]	R^2
Stenosporic acid	69.48	61.14	78.96	0.95

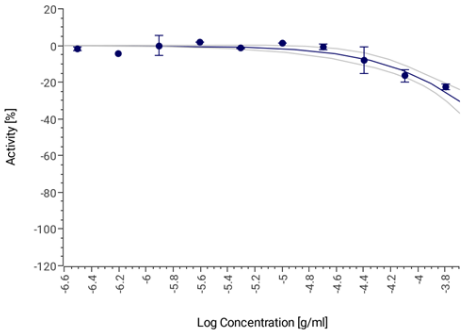

Compound ID	qAC50 [µg/mL]	Lower 95% CL [µg/mL]	Upper 95% CL [µg/mL]	R^2
Sekikaic acid	> 160	-	-	0.82

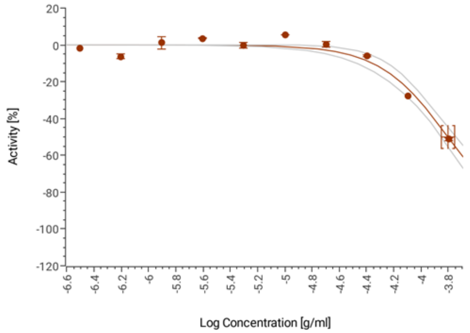

Compound ID	qAC50 [µg/mL]	Lower 95% CL [µg/mL]	Upper 95% CL [µg/mL]	R^2
4'-O-methylpaludosic acid	157.7	-	-	0.94

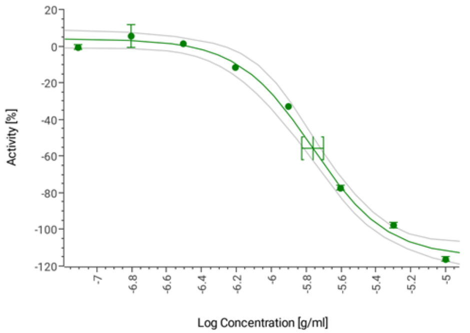

Compound ID	qAC50 [g/ml]	Lower 95% CL [g/ml]	Upper 95% CL [g/ml]	R^2
Amphotericin B	1.73	1.50	1.98	0.99

Figure S30. Dose Response Curves, EC50 values and their corresponding 95% confidence limits for compounds 1-5 and Amphotericin B obtained from *C. acutatum* absorbance-based assay.

Colletotrichum acutatum Fluorescence Based Assay

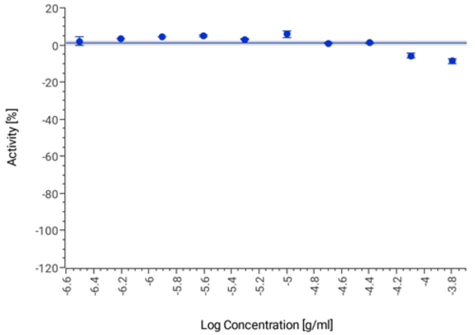

Compound ID	qAC50 [µg/mL]	Lower 95% CL [µg/mL]	Upper 95% CL [µg/mL]	R^2
8-hydroxyfumarprotocetraric acid	> 160	-	-	-

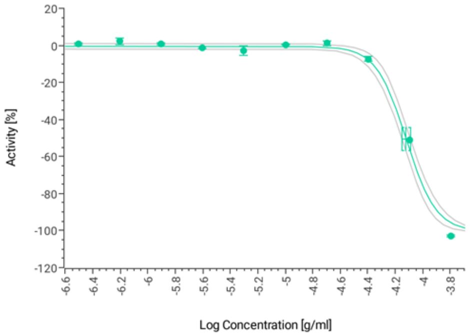

Compound ID	qAC50 [µg/mL]	Lower 95% CL [µg/mL]	Upper 95% CL [µg/mL]	R^2
Divaricatic acid	75.75	70.59	81.29	0.99

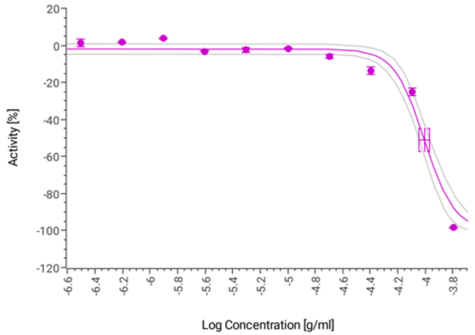

Compound ID	qAC50 [µg/mL]	Lower 95% CL [µg/mL]	Upper 95% CL [µg/mL]	R^2
Stenosporic acid	98.49	90.32	107.4	0.95

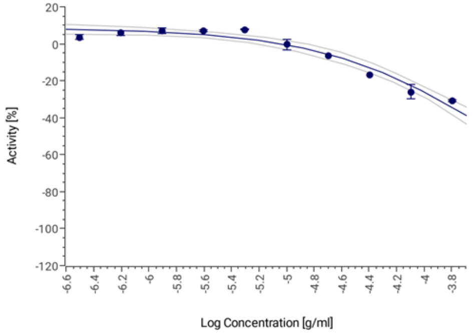

Compound ID	qAC50 [µg/mL]	Lower 95% CL [µg/mL]	Upper 95% CL [µg/mL]	R^2
Sekikaic acid	> 160	-	-	0.94

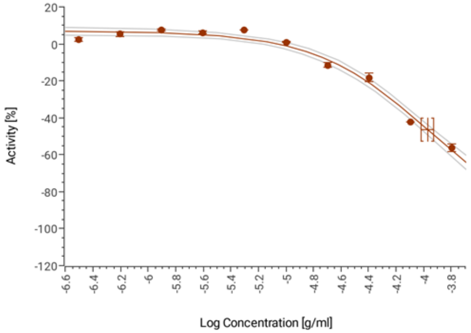

Compound ID	qAC50 [µg/mL]	Lower 95% CL [µg/mL]	Upper 95% CL [µg/mL]	R^2
4'-O-methylpaludosic acid	106.8	96.37	118.3	0.97

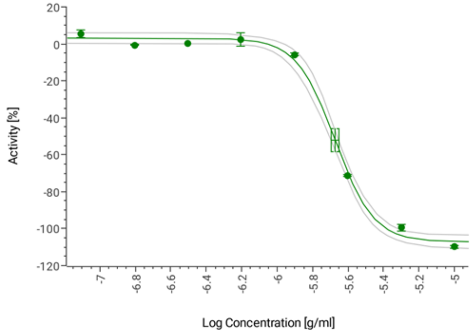

Compound ID	qAC50 [g/ml]	Lower 95% CL [g/ml]	Upper 95% CL [g/ml]	R^2
Amphotericin B	2.12	2.01	2.24	0.99

Figure S31. Dose Response Curves, EC50 values and their corresponding 95% confidence limits for compounds 1-5 and Amphotericin B obtained from *C. acutatum* fluorescence-based assay.

Fusarium oxysporum TR4 Absorbance Based Assay

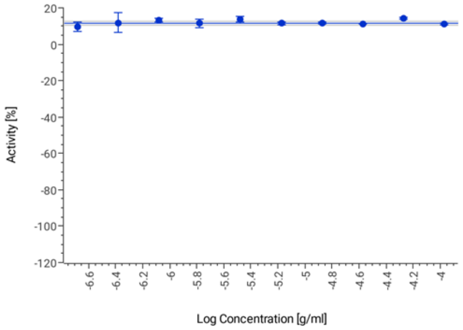

Compound ID	qAC50 [µg/mL]	Lower 95% CL [µg/mL]	Upper 95% CL [µg/mL]	R^2
8-hydroxyfumarprotocetraric acid	> 106.7	-	-	-

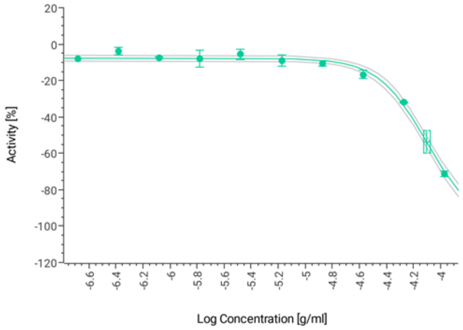

Compound ID	qAC50 [µg/mL]	Lower 95% CL [µg/mL]	Upper 95% CL [µg/mL]	R^2
Divaricatic acid	79.00	74.76	83.47	0.98

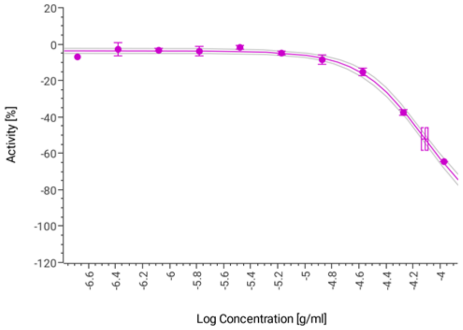

Compound ID	qAC50 [µg/mL]	Lower 95% CL [µg/mL]	Upper 95% CL [µg/mL]	R^2
Stenosporic acid	77.02	73.03	81.23	0.99

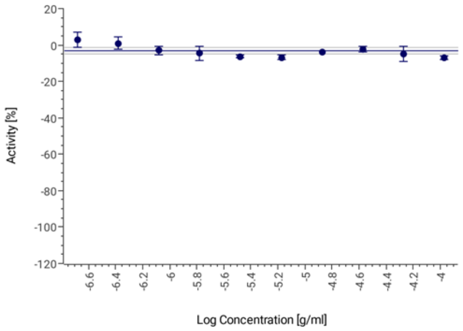

Compound ID	qAC50 [µg/mL]	Lower 95% CL [µg/mL]	Upper 95% CL [µg/mL]	R^2
Sekikaic acid	> 106.7	-	-	-

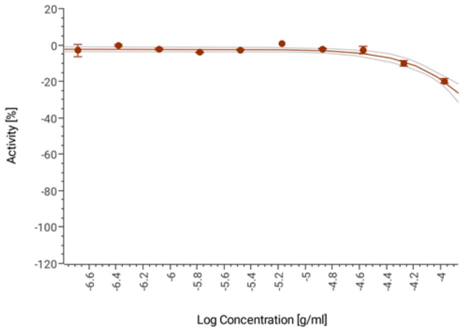

Compound ID	qAC50 [µg/mL]	Lower 95% CL [µg/mL]	Upper 95% CL [µg/mL]	R^2
4'-O-methylpaludosic acid	> 106.7	-	-	0.87

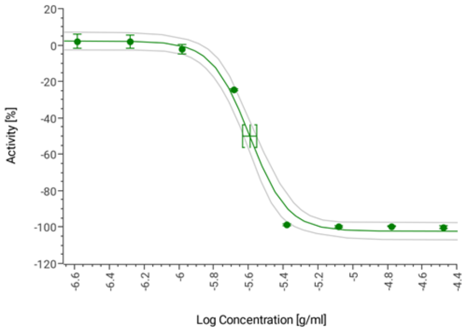

Compound ID	qAC50 [g/ml]	Lower 95% CL [g/ml]	Upper 95% CL [g/ml]	R^2
Amphotericin B	2.56	2.32	2.81	0.99

Figure S32. Dose Response Curves, EC50 values and their corresponding 95% confidence limits for compounds 1-5 and Amphotericin B obtained from *F. oxysporum TR4* absorbance-based assay.

Fusarium oxysporum TR4 Fluorescence Based Assay

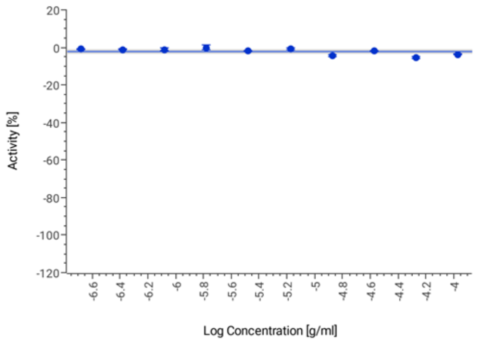

Compound ID	qAC50 [µg/mL]	Lower 95% CL [µg/mL]	Upper 95% CL [µg/mL]	R^2
8-hydroxyfumarprotocetraric acid	> 106.7	-	-	-

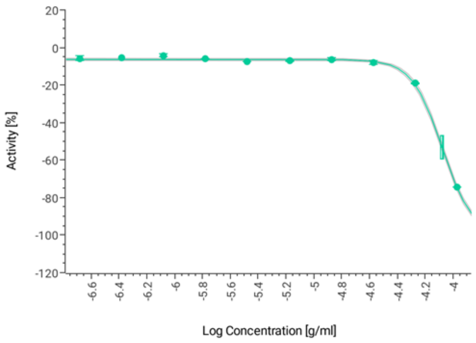

Compound ID	qAC50 [µg/mL]	Lower 95% CL [µg/mL]	Upper 95% CL [µg/mL]	R^2
Divaricatic acid	83.28	81.79	84.80	0.99

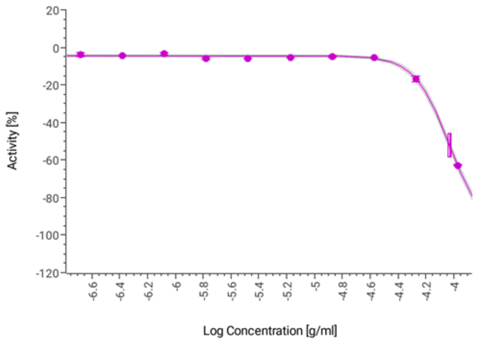

Compound ID	qAC50 [µg/mL]	Lower 95% CL [µg/mL]	Upper 95% CL [µg/mL]	R^2
Stenosporic acid	93.47	91.55	95.42	0.99

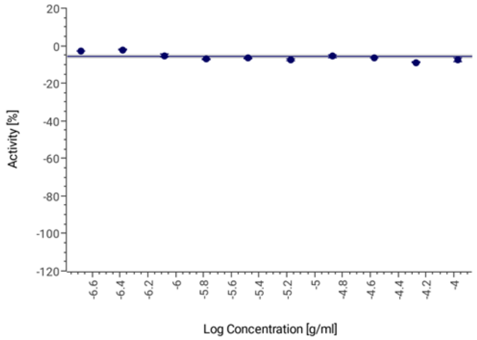

Compound ID	qAC50 [µg/mL]	Lower 95% CL [µg/mL]	Upper 95% CL [µg/mL]	R^2
Sekikaic acid	> 106.7	-	-	-

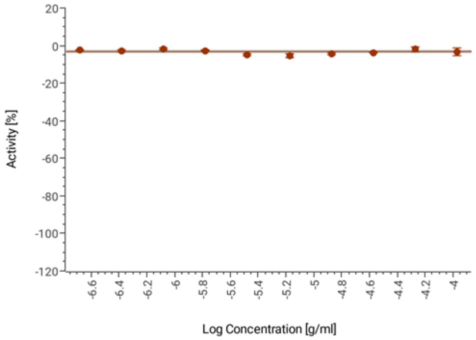

Compound ID	qAC50 [µg/mL]	Lower 95% CL [µg/mL]	Upper 95% CL [µg/mL]	R^2
4'-O-methylpaludosic acid	> 106.7	-	-	-

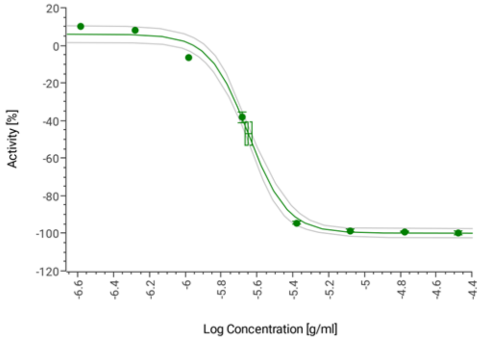

Compound ID	qAC50 [g/ml]	Lower 95% CL [g/ml]	Upper 95% CL [g/ml]	R^2
Amphotericin B	2.25	2.14	2.35	0.99

Figure S33. Dose Response Curves, EC50 values and their corresponding 95% confidence limits for compounds 1-5 and Amphotericin B obtained from *F. oxysporum TR4* fluorescence-based assay.

Magnaporthe grisea Absorbance Based Assay

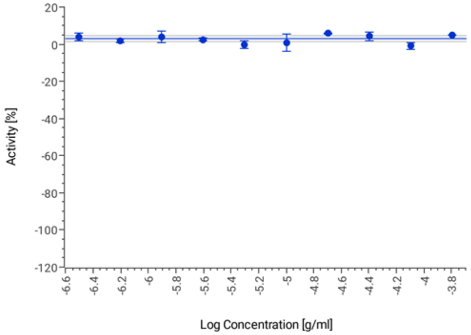

Compound ID	qAC50 [µg/mL]	Lower 95% CL [µg/mL]	Upper 95% CL [µg/mL]	R^2
8-hydroxyfumarprotocetraric acid	> 160	-	-	-

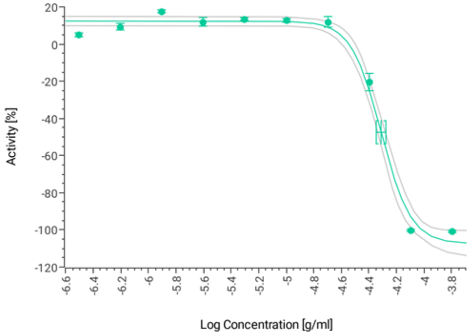

Compound ID	qAC50 [µg/mL]	Lower 95% CL [µg/mL]	Upper 95% CL [µg/mL]	R^2
Divaricatic acid	48.85	45.14	52.86	0.98

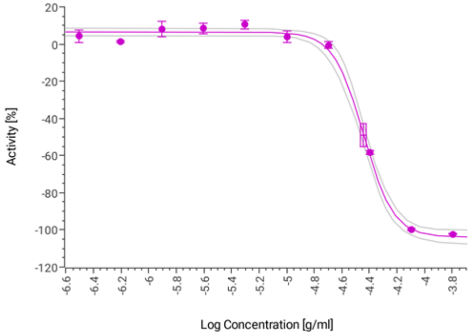

Compound ID	qAC50 [µg/mL]	Lower 95% CL [µg/mL]	Upper 95% CL [µg/mL]	R^2
Stenosporic acid	36.06	34.37	37.85	0.99

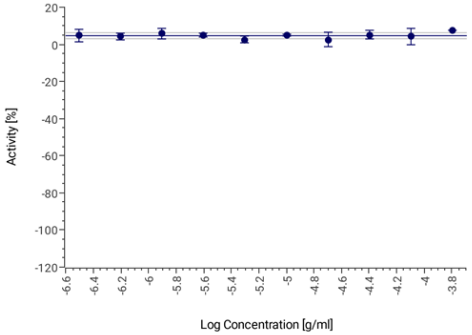

Compound ID	qAC50 [µg/mL]	Lower 95% CL [µg/mL]	Upper 95% CL [µg/mL]	R^2
Sekikaic acid	> 160	-	-	-

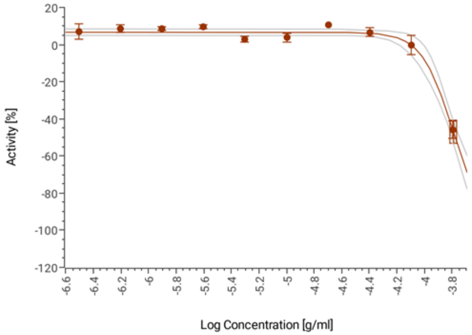

Compound ID	qAC50 [µg/mL]	Lower 95% CL [µg/mL]	Upper 95% CL [µg/mL]	R^2
4'-O-methylpaludosic acid	> 160	-	-	0.94

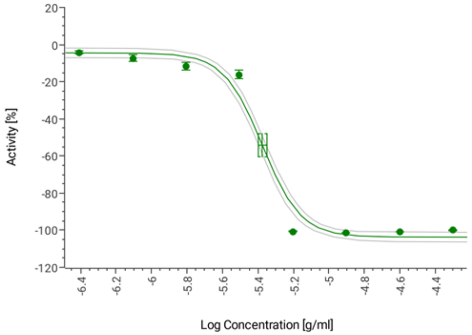

Compound ID	qAC50 [g/ml]	Lower 95% CL [g/ml]	Upper 95% CL [g/ml]	R^2
Amphotericin B	4.21	3.98	4.45	0.97

Figure S34. Dose Response Curves, EC50 values and their corresponding 95% confidence limits for compounds 1-5 and Amphotericin B obtained from *M. grisea* absorbance-based assay.

Magnaporthe grisea Fluorescence Based Assay

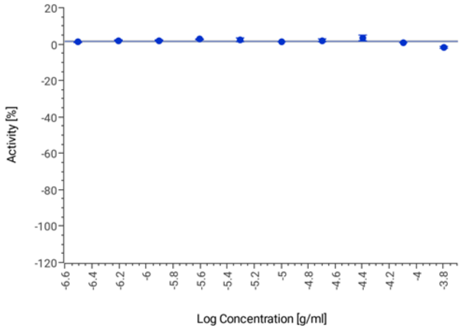

	Compound ID	qAC50 [µg/mL]	Lower 95% CL [µg/mL]	Upper 95% CL [µg/mL]	R^2
■	8-hydroxyfumarprotocetraric acid	> 160	-	-	-

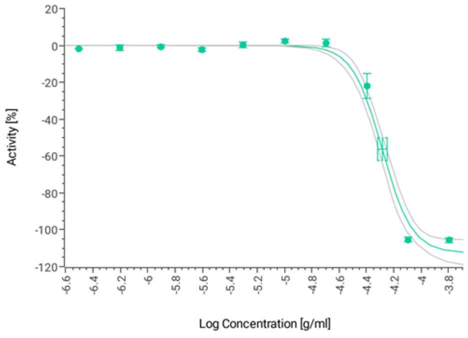

	Compound ID	qAC50 [µg/mL]	Lower 95% CL [µg/mL]	Upper 95% CL [µg/mL]	R^2
■	Divaricatic acid	51.85	47.64	56.43	0.98

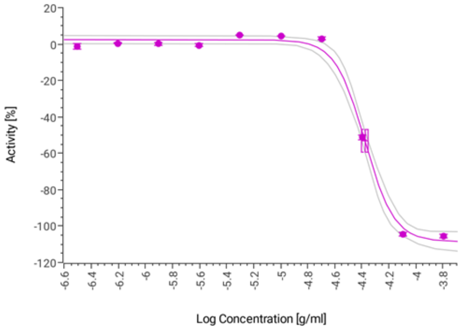

	Compound ID	qAC50 [µg/mL]	Lower 95% CL [µg/mL]	Upper 95% CL [µg/mL]	R^2
■	Stenosporic acid	41.88	39.39	44.53	0.99

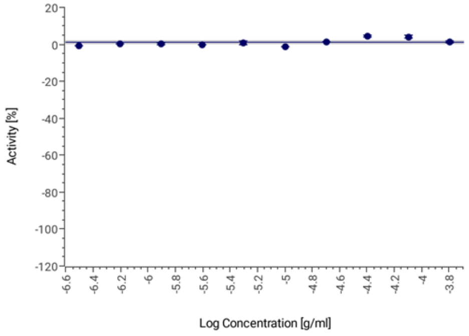

	Compound ID	qAC50 [µg/mL]	Lower 95% CL [µg/mL]	Upper 95% CL [µg/mL]	R^2
■	Sekikaic acid	> 160	-	-	-

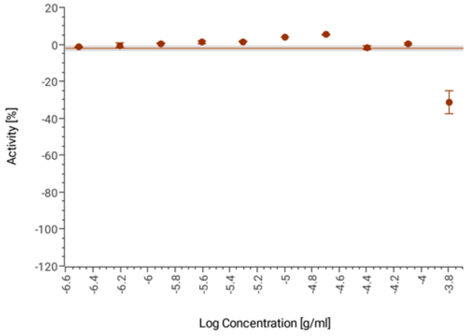

	Compound ID	qAC50 [µg/mL]	Lower 95% CL [µg/mL]	Upper 95% CL [µg/mL]	R^2
■	4'-O-methylpaludosic acid	> 160	-	-	-

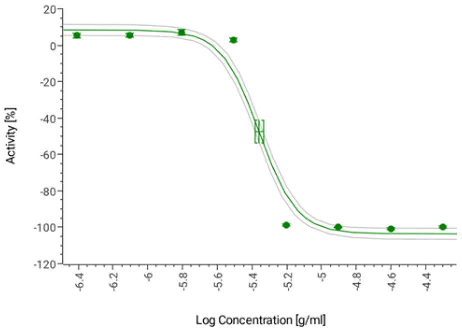

	Compound ID	qAC50 [g/ml]	Lower 95% CL [g/ml]	Upper 95% CL [g/ml]	R^2
■	Amphotericin B	4.38	4.13	4.64	0.97

Figure S35. Dose Response Curves, EC50 values and their corresponding 95% confidence limits for compounds 1-5 and Amphotericin B obtained from *M. grisea* fluorescence-based assay.

Verticillium dahliae Absorbance Based Assay

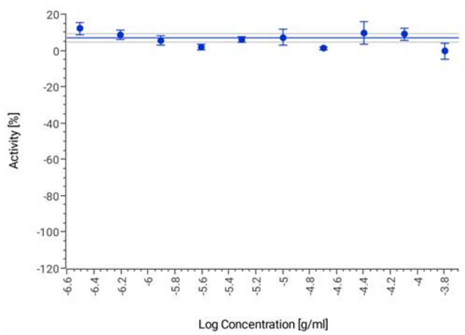

	Compound ID	qAC50 [µg/mL]	Lower 95% CL [µg/mL]	Upper 95% CL [µg/mL]	R^2
■	8-hydroxyfumarprotocetraric acid	> 160	-	-	-

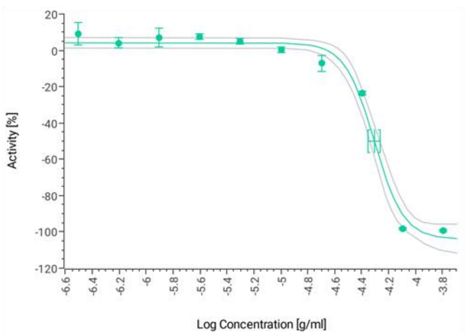

	Compound ID	qAC50 [µg/mL]	Lower 95% CL [µg/mL]	Upper 95% CL [µg/mL]	R^2
■	Divaricatic acid	49.38	44.52	54.77	0.98

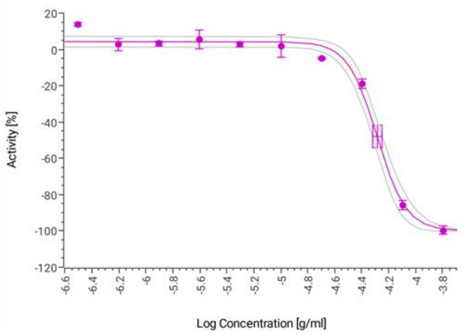

	Compound ID	qAC50 [µg/mL]	Lower 95% CL [µg/mL]	Upper 95% CL [µg/mL]	R^2
■	Stenosporic acid	51.75	47.67	56.17	0.97

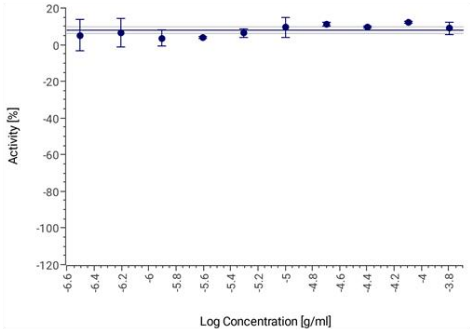

	Compound ID	qAC50 [µg/mL]	Lower 95% CL [µg/mL]	Upper 95% CL [µg/mL]	R^2
■	Sekikaic acid	> 160	-	-	-

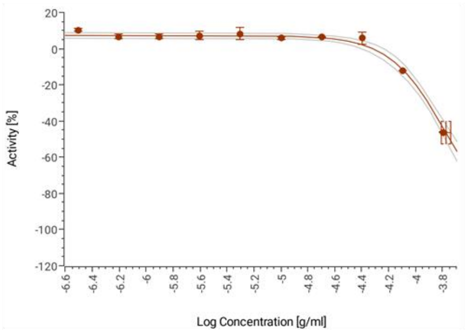

	Compound ID	qAC50 [µg/mL]	Lower 95% CL [µg/mL]	Upper 95% CL [µg/mL]	R^2
■	4'-O-methylpaludosic acid	> 160	-	-	0.96

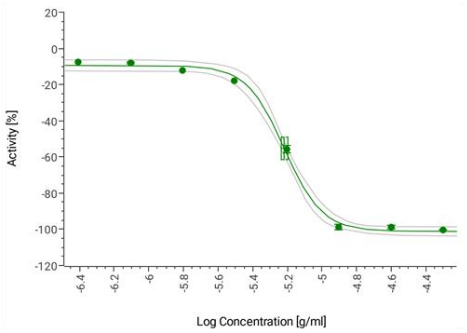

	Compound ID	qAC50 [g/ml]	Lower 95% CL [g/ml]	Upper 95% CL [g/ml]	R^2
■	Amphotericin B	6.07	5.79	6.35	0.99

Figure S36. Dose Response Curves, EC50 values and their corresponding 95% confidence limits for compounds 1-5 and Amphotericin B obtained from *V. dahliae* absorbance-based assay.

Verticillium dahliae Fluorescence Based Assay

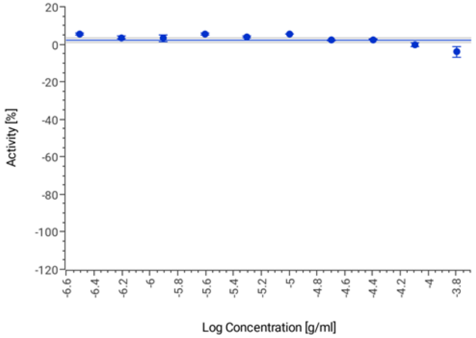

Compound ID	qAC50 [µg/mL]	Lower 95% CL [µg/mL]	Upper 95% CL [µg/mL]	R^2
8-hydroxyfumarprotocetraric acid	> 160	-	-	-

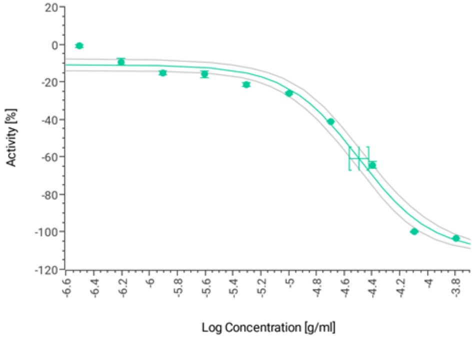

Compound ID	qAC50 [µg/mL]	Lower 95% CL [µg/mL]	Upper 95% CL [µg/mL]	R^2
Divaricatic acid	32.11	27.45	37.58	0.97

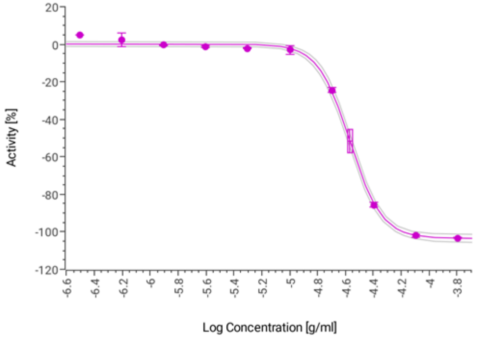

Compound ID	qAC50 [µg/mL]	Lower 95% CL [µg/mL]	Upper 95% CL [µg/mL]	R^2
Stenosporic acid	27.03	25.98	28.11	0.99

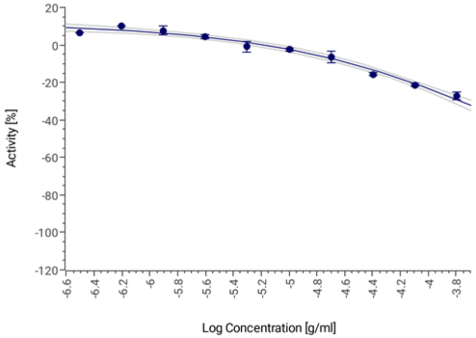

Compound ID	qAC50 [µg/mL]	Lower 95% CL [µg/mL]	Upper 95% CL [µg/mL]	R^2
Sekikaic acid	> 160	-	-	0.97

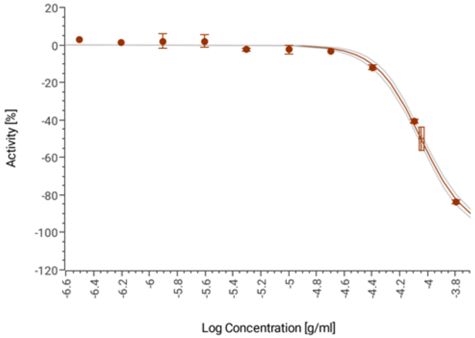

Compound ID	qAC50 [µg/mL]	Lower 95% CL [µg/mL]	Upper 95% CL [µg/mL]	R^2
4'-O-methylpaludosic acid	89.78	86.31	93.39	0.99

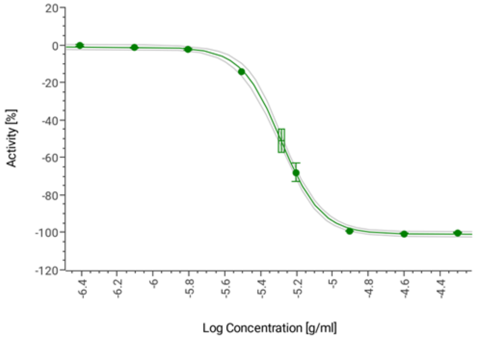

Compound ID	qAC50 [g/ml]	Lower 95% CL [g/ml]	Upper 95% CL [g/ml]	R^2
Amphotericin B	5.19	5.01	5.38	0.99

Figure S37. Dose Response Curves, EC50 values and their corresponding 95% confidence limits for compounds 1-5 and Amphotericin B obtained from *V. dahliae* fluorescence-based assay.

Fusarium proliferatum Absorbance Based Assay

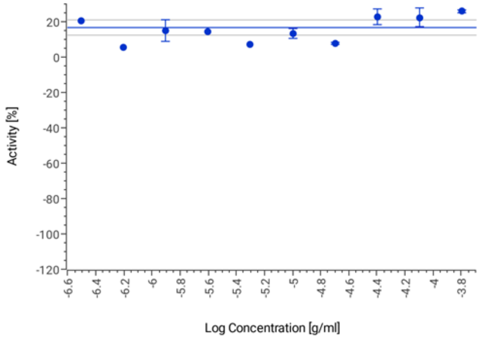

Compound ID	qAC50 [µg/mL]	Lower 95% CL [µg/mL]	Upper 95% CL [µg/mL]	R^2
8-hydroxyfumarprotocetraric acid	> 160	-	-	-

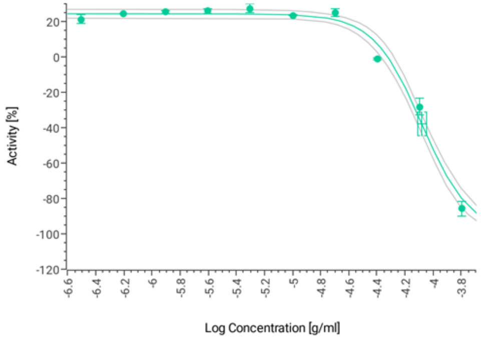

Compound ID	qAC50 [µg/mL]	Lower 95% CL [µg/mL]	Upper 95% CL [µg/mL]	R^2
Divaricatic acid	83.13	77.55	89.12	0.98

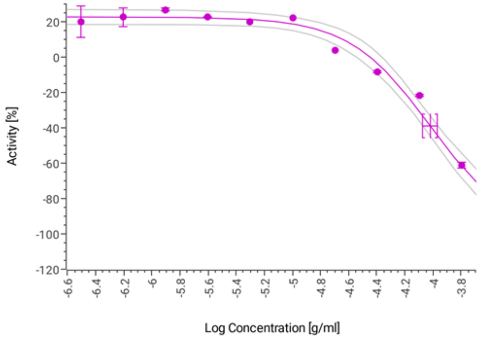

Compound ID	qAC50 [µg/mL]	Lower 95% CL [µg/mL]	Upper 95% CL [µg/mL]	R^2
Stenosporic acid	94.99	83.70	107.8	0.96

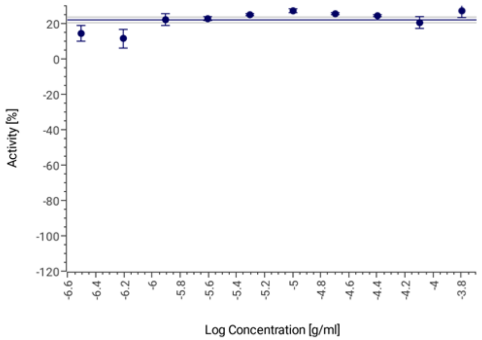

Compound ID	qAC50 [µg/mL]	Lower 95% CL [µg/mL]	Upper 95% CL [µg/mL]	R^2
Sekikaic acid	> 160	-	-	-

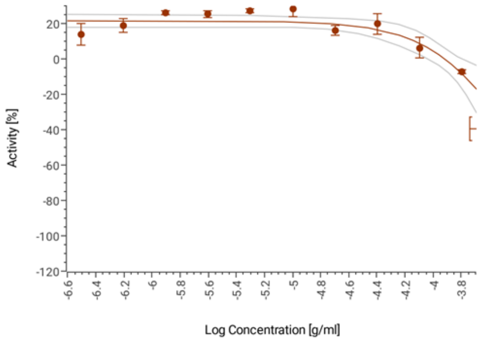

Compound ID	qAC50 [µg/mL]	Lower 95% CL [µg/mL]	Upper 95% CL [µg/mL]	R^2
4'-O-methylpaludosic acid	> 160	-	-	0.63

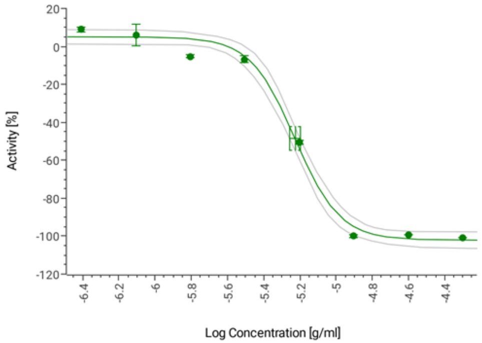

Compound ID	qAC50 [g/ml]	Lower 95% CL [g/ml]	Upper 95% CL [g/ml]	R^2
Amphotericin B	5.93	5.51	6.38	0.99

Figure S38. Dose Response Curves, EC50 values and their corresponding 95% confidence limits for compounds 1-5 and Amphotericin B obtained from *F. proliferatum* absorbance-based assay.

Fusarium proliferatum Fluorescence Based Assay

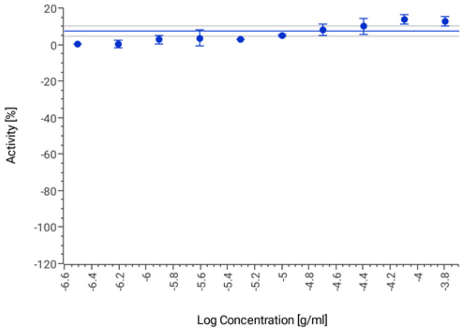

Compound ID	qAC50 [µg/mL]	Lower 95% CL [µg/mL]	Upper 95% CL [µg/mL]	R^2
8-hydroxyfumarprotocetraric acid	> 160	-	-	-

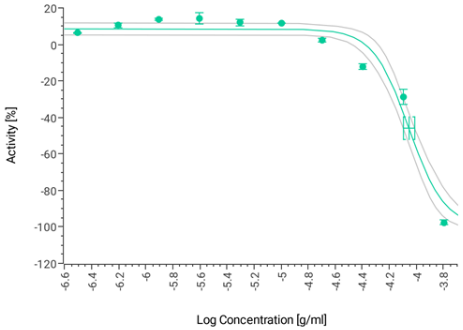

Compound ID	qAC50 [µg/mL]	Lower 95% CL [µg/mL]	Upper 95% CL [µg/mL]	R^2
Divaricatic acid	88.07	80.20	96.70	0.95

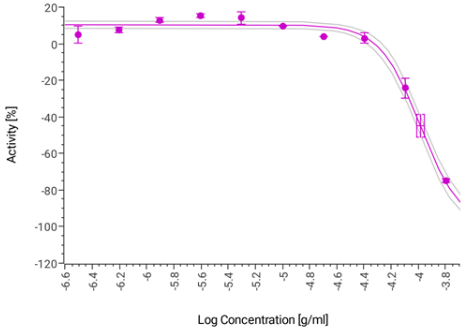

Compound ID	qAC50 [µg/mL]	Lower 95% CL [µg/mL]	Upper 95% CL [µg/mL]	R^2
Stenosporic acid	103.3	97.17	109.70	0.97

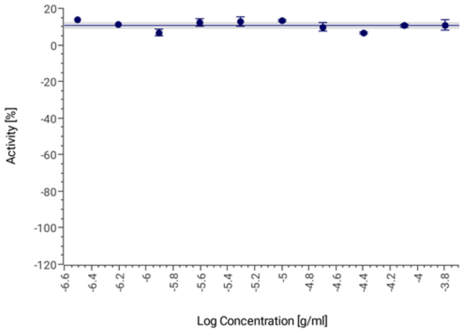

Compound ID	qAC50 [µg/mL]	Lower 95% CL [µg/mL]	Upper 95% CL [µg/mL]	R^2
Sekikaic acid	> 160	-	-	-

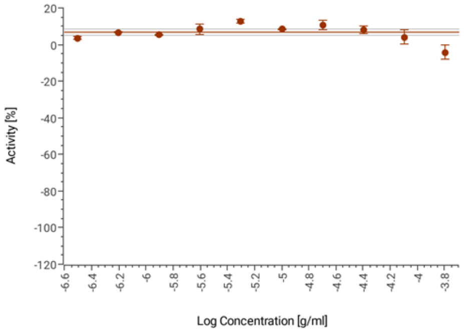

Compound ID	qAC50 [µg/mL]	Lower 95% CL [µg/mL]	Upper 95% CL [µg/mL]	R^2
4'-O-methylpaludosic acid	> 160	-	-	-

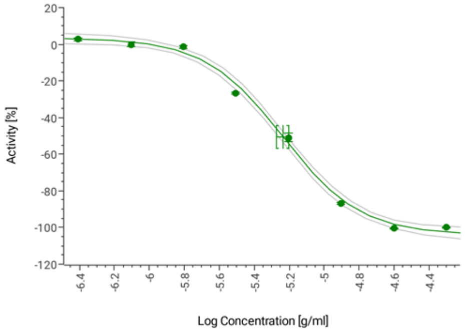

Compound ID	qAC50 [g/ml]	Lower 95% CL [g/ml]	Upper 95% CL [g/ml]	R^2
Amphotericin B	5.78	5.35	6.25	0.99

Figure S39. Dose Response Curves, EC50 values and their corresponding 95% confidence limits for compounds 1-5 and Amphotericin B obtained from *F. proliferatum* fluorescence-based assay.
